# Genome-Wide Identification of *DnaJ* Gene Family and VIGS Analysis Reveal the Function of *GhDnaJ316* in Floral Development for Upland Cotton

**DOI:** 10.3390/plants14213380

**Published:** 2025-11-05

**Authors:** Ting-Ting Zhang, Xue-Feng Guo, Dan-Dan Li, Yun Jia, Chen-Hui Wang, Yu-Nuo Fan, Cai-Xiang Wang, Jun-Ji Su

**Affiliations:** 1 State Key Laboratory of Aridland Crop Science, College of Life Science and Technology, Gansu Agricultural University, Lanzhou 730070, China; guoxuefeng2025@163.com (X.-F.G.); m15293180496@163.com (D.-D.L.); wangchenhui1201@163.com (C.-H.W.); fanyn695@163.com (Y.-N.F.); wangcaix@gsau.edu.cn (C.-X.W.); 2 Xi’an Botanical Garden of Shaanxi Province (Institute of Botany of Shaanxi Province), Xi’an 710061, China; jiayun@xab.ac.cn

**Keywords:** *DnaJ* gene, expression pattern, floral development, upland cotton, VIGS

## Abstract

DnaJ proteins are established regulators of multiple physiological processes in plants, but their systematic identification and functional characterization in cotton remains largely uncharacterized, particularly regarding their roles in floral developmental regulation. In this study, based on genome-wide analysis of *Gossypium hirsutum* L., 372 *DnaJ* genes were systematically identified and phylogenetically classified into four distinct clades (I–IV). These genes exhibited non-uniform chromosomal distribution. Structural analysis revealed clade-specific variations in intron numbers and conserved motifs. Cis-acting element profiling indicated the roles of *DnaJs* in modulating biosynthetic and metabolic regulation during both vegetative and reproductive development in cotton. Transcriptomic analysis highlighted tissue-specific expression patterns, with *GhDnaJ316* showing preferential expression in anthers and filaments. Functional validation via VIGS-mediated silencing confirmed *GhDnaJ316* as a negative regulator of floral transition, accelerating budding by 7.7 days and flowering by 9.7 days in silenced plants. This study elucidates the genomic architecture of *GhDnaJs*, demonstrates *GhDnaJ316*’s critical role in floral development and provides insights for molecular breeding in early-maturing cotton.

## 1. Introduction

DnaJ proteins, also referred to as J-proteins, constitute the conserved family of co-chaperones characterized by the presence of a highly conserved J-domain. DnaJ protein was ubiquitously distributed across eukaryotes, initially identified as the 41 kDa heat shock protein in the study on *Escherichia coli* heat shock response mechanism [1]. DnaJ proteins exhibit remarkable diversity in both quantity and structural organization. Based on domain architecture, they were historically classified into three categories, i.e., Type I, II, and III. Type I consisted of four domains including the J-domain, a zinc finger domain, a glycine/phenylalanine-rich region (G/F domain), and a less conserved C-terminal domain. Type II harbored the J-domain and G/F domain without zinc finger domain. Type III was the most minimalistic, consisting solely of the J-domain [2,3,4,5]. Recently, Type IV DnaJs have been identified as a new category characterized by a domain structurally analogous to the J-domain but lacking the complete signature His, Pro and Asp (HPD) tripeptide motifs [6].

Accumulating evidence indicates that DnaJ proteins, functioning as molecular co-chaperones of Hsp70 systems, orchestrate diverse physiological processes including seed germination, chloroplast biogenesis, photosynthetic efficiency, protein trafficking, anther morphogenesis, flowering time control, seed maturation, and abiotic stress response [7,8,9,10]. For instance, Vitha et al. demonstrated in *Arabidopsis thaliana* that loss-of-function mutations in the plastid-localized J-protein ARC6 disrupt the formation of the chloroplast division machinery, resulting in severely impaired plastid fission due to defective Z-ring assembly at the mid-plastid site [11]. Studies have revealed that mutations in chloroplast-localized J-proteins J8, J11, and J20 in *A*. *thaliana* significantly impair photosynthetic enzyme activity and destabilize the assembly of the photosystem II (PSII) complex, demonstrating their critical roles in regulating photosynthetic efficiency [12,13,14,15]. Furthermore, mitochondria-localized DnaJ proteins were found to recruit Hsp70 chaperones to the inner membrane through direct protein–protein interactions, thereby mediating the translocation of cytosolic polypeptides into the mitochondrial matrix [8].

*DnaJ* genes have been identified as key regulators in floral organ development and flowering time modulation in model plants [16,17]. For example, the J-protein ERdj3B maintains its chaperone function under heat stress to promote anther development in *A. thaliana* by facilitating the assembly of the SDF2-ERdj3B-BiP complex, which ensures proper protein folding in reproductive tissues [18]. Furthermore, *AtJ1* accelerates flowering initiation through negative regulation of ABA signaling pathways [19]. Yang et al. demonstrated that *CbuDnaJ49* modulates leaf pigmentation in *Catalpa bungei* by regulating anthocyanin biosynthesis and chloroplast development [20]. The *DnaJ* gene family was also found to participate in plant responses to heat, drought and salinity stresses through distinct molecular mechanisms [21,22]. In *Arabidopsis thaliana*, *AtDjB1* acts as a negative regulator of drought stress adaptation by suppressing ABA-mediated stomatal closure and root architecture remodeling, thereby exacerbating water loss under dehydration conditions [23]. Conversely, in *Oryza sativa*, the nuclear-localized *OsDnaJ15* chaperone enhances salt tolerance by forming a complex with *OsBAG4*; the maintenance of this ion homeostasis maintains the efficiency of both photochemical reactions and carbon fixation during photosynthesis, enabling sustained photosynthetic capacity under saline stress [24]. Nevertheless, the regulatory functions of the DnaJ protein family in floral transition, particularly in economically important crops, have not been extensively investigated.

Cotton serves as a fundamental pillar of agricultural economy worldwide and a critical strategic resource, with upland cotton (*Gossypium hirsutum* L.) accounting for 95% of global production and representing the dominant cultivar in cotton breeding [25,26]. The development of sequencing technologies and multi-omics research have progressively elucidated genes regulating growth and development in *Gossypium* species [27,28,29]. For instance, integrated RTM-GWAS and meta-QTL analyses identified two genomic regions significantly associated with the node of the first fruiting branch (FFBN) and its height (HFFBN), pinpointing candidate genes *GhE6*, *GhDRM1*, and *GhGES*. Subsequent VIGS validation confirmed the regulation of *GhE6* in modulating HFFBN, while *GhDRM1* and *GhGES* were linked to early flowering [30]. Wang et al. (2023) [31] revealed that *GhAP1-D3* positively regulates flowering time and early maturity, overexpression assays revealed that this gene significantly accelerates flowering and shortens the maturation period without compromising yield or fiber quality. Furthermore, studies on the transcription factor *GhNST1* indicate its upregulated expression under drought stress, and VIGS-based silencing of the gene reduced relative water content, increased wilting rates, diminished antioxidant enzyme activity, and delayed bud emergence, flowering, and boll opening. Concurrently, stress-responsive genes (e.g., *GhDREB2A*) were downregulated, underscoring that *GhNST1* as a key integrator of drought resistance and developmental regulation [32]. Collectively, these studies highlight the potential of mining and characterizing early-maturity-related genes in upland cotton to enrich the genetic foundation for molecular marker development and the breeding of early-maturing and superior-quality cotton germplasm. In this study, we conducted a comprehensive phylogenetic analysis of the *DnaJs* gene family to delineate evolutionary conservation and diversification patterns in upland cotton, followed by the investigation of conserved protein motifs, gene structure, and promoter cis-acting elements. Moreover, Tissue-specific expression profiles of *GhDnaJs* were characterized to understand its expression characteristics. Subsequently, *GhDnaJ316* was functionally validated through virus-induced gene silencing (VIGS), revealing its regulatory role in floral development. This study provides potential gene resources for molecular breeding in cotton.

## 2. Results

### 2.1. Identification and Physiochemical Properties of GhDnaJs

Bioinformatic analysis of the *G. hirsutum* genome identified a total of 372 candidate *DnaJ* genes. These genes were systematically designated as *GhDnaJ01* to *GhDnaJ372* based on their chromosomal locations for convenience (Table S2). Physicochemical characterization revealed that DnaJ proteins exhibit an average molecular weight of 52.68 kDa. Their isoelectric points (pI) ranged from 4.49 to 11.29, with instability indices spanning 19.03 to 79.03. Among them, approximately 55% of the DnaJ protein family exhibited pI values exceeding 7, indicating a balanced distribution of acidic and basic isoforms within *G. hirsutum* (Table S3). Subcellular localization predictions indicated that DnaJ proteins were primarily localized to the nucleus (44.62%), chloroplasts (21.77%), cytoplasm (16.67), and plasma membrane (8.87%), with minor distributions observed in the mitochondrion (3.49%), vacuolar membrane (1.61%), peroxisome (1.08%), cytoskeleton (1.08%), extracellular (0.54%), and endoplasmic reticulum (0.27%) (Figure S1).

### 2.2. Phylogenetic Tree Analysis

**Figure 1:**
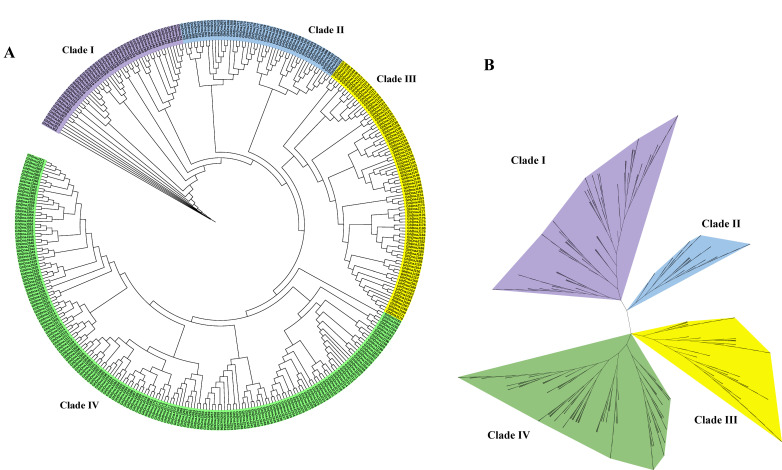
Phylogenetic relationships of the 372 DnaJ proteins in *G*. *hirsutum*. (**A**) Circular phylogenetic tree. (**B**) Radial phylogenetic tree diagram.

To elucidate phylogenetic relationships and classification patterns within the *GhDnaJ* gene family, we employed 372 protein sequences from *G. hirsutum* to reconstruct an unrooted phylogenetic tree based on multiple sequence alignment (Figure 1). To delineate the clustering relationships within the phylogenetic tree with enhanced clarity, we generated circular and radial phylogenetic tree diagrams. The phylogenetic topology from both circular and radial tree suggested that the *DnaJ* gene family in *G*. *hirsutum* diverged into four major clades (Clade I, II, III, and IV), comprising 53, 50, 86, and 183 members, respectively, indicating a primary classification framework comprising four distinct subfamilies. Additionally, the reconstructed phylogenetic tree incorporating DnaJ protein sequences from *G. hirsutum* and *A. thaliana* exhibited obvious similarity in topological structure to the tree generated exclusively from *G. hirsutum* DnaJ sequences, consistently supporting the division of the DnaJ family into four evolutionary clades (Figure S2).

### 2.3. Gene Structure and Conserved Domain

**Figure 2:**
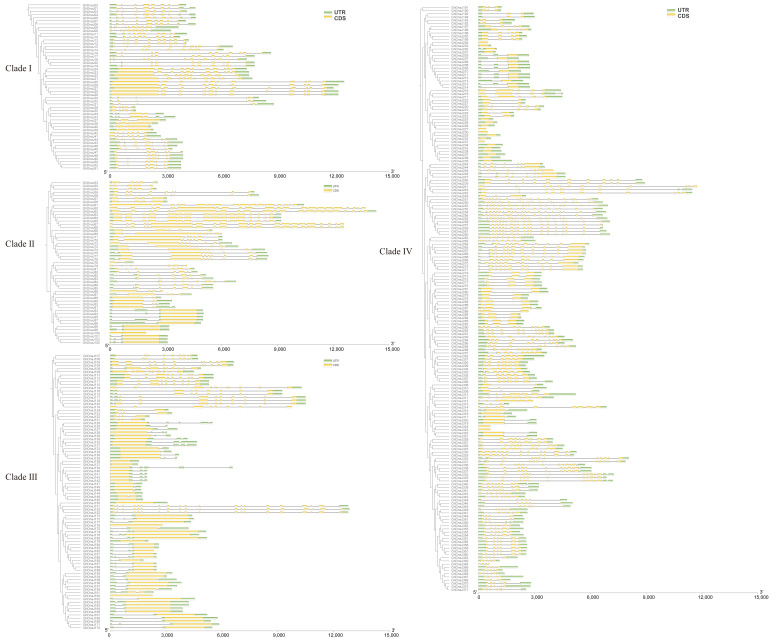
Structural analysis of *GhDnaJ* genes within individual phylogenetic clades. Green bars represent untranslated regions (UTRs), while yellow bars denote coding sequences (CDSs).

The intron–exon architecture, intron classification, and intron density collectively represent typical phylogenetic signatures within gene families. Comparative analysis of intron–exon architectures across 372 *GhDnaJ* genes revealed conserved structural patterns within individual phylogenetic clades, whereas significant divergences were observed among distinct evolutionary branches (Figure 2). Intron count across the *GhDnaJ* gene family exhibited a range of 1 to 22, with a minority of genes (1–2 introns) classified as low-intron variants. Clade-specific analysis revealed distinct distribution patterns: genes in Clade I consistently contained 4–10 introns, whereas Clades II, III, and IV displayed broader variation (1–21, 1–19, and 1–22 introns, respectively). The predicted conserved motifs of *DnaJ* genes corroborated the evolutionary classification established by *DnaJs* family phylogenetic analysis. The sequence features and amino acid lengths of conserved motifs are shown in Figure S3, with Motifs 1–4 and Motif 9 identified as structurally conserved *DnaJ* domains across all analyzed *DnaJ* proteins. Members of the *DnaJ* gene family on evolutionary clade I contain one to five motifs, whereas those on clades II, III, and IV all possess Motifs 8, 10,11, and 14. The prediction of these motifs demonstrates that the *DnaJ* gene family exhibits relative functional and structural conservation among members within the same evolutionary clade.

### 2.4. Chromosome Location, Gene Structures, and Cis-Acting Elements

**Figure 3:**
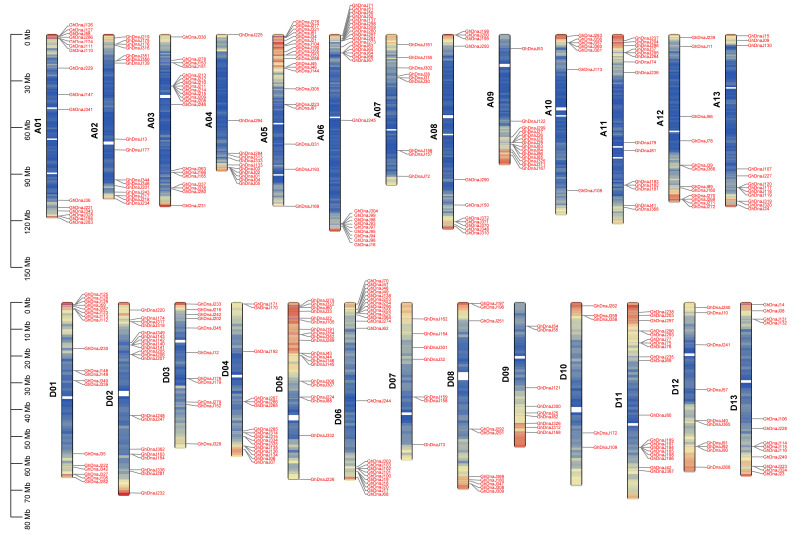
Chromosome location of *GhDnaJ* genes across the A and D subgenomes. Red color indicates the positions of *GhDnaJs* gene family members on the chromosomes. The color on the chromosomes transitions from red to blue, with red representing regions of dense *GhDnaJs* gene distribution. Yellow represents regions of scattered *GhDnaJs* gene distribution, and blue represents regions with no *GhDnaJs* gene distribution. The A06 chromosome harbored the most quantity of *GhDnaJ* genes, reaching 26, while D10 contained the fewest with a number of 5.

To investigate the distribution and structural characteristics of the *DnaJ* gene family, chromosomal localization and gene structure analysis were performed. The results revealed that 372 *DnaJ* genes in *G. hirsutum* are predominantly distributed at the terminal regions of chromosomes. The A06 chromosome harbored the highest number of *DnaJ* genes, totaling 26, whereas D10 chromosomes exhibit the lowest *DnaJ* gene content, with 5 genes, indicating an uneven distribution of the *DnaJ* family across the A and D subgenomes (Figure 3). Secondary structure prediction demonstrated that DnaJ proteins in *G. hirsutum* primarily consist of α-helices, β-sheets, and random coils, accounting for 38.30%, 7.51%, and 54.19% of the structural composition, respectively (Figure S4). Tertiary structure analysis of representative genes from major clades in the phylogenetic tree further revealed distinct structural patterns: Clade I and Clade II members exhibited prominent α-helix content and a small amount of random coils; Clade III and Clade IV members were dominated by random coils except for several GhDnaJ proteins (Figure S5). This demonstrated that clade-specific divergence in both secondary and tertiary folds, likely facilitate the subfunctionalization within *DnaJs* family during polyploid evolution.

Multiple cis-acting elements were identified within the 2000-bp upstream sequences of the transcription start sites of *DnaJ* genes in *G*. *hirsutum*, encompassing defense and stress-responsive elements (such as drought induced, wound response, low temperature response, and anaerobic induction), phytohormone-responsive elements (including abscisic acid response, gibberellin response, auxin response, salicylic acid response, and zein metabolism regulation), binding sites (e.g., AT-rich DNA binding protein, MYBHv1 binding site, and conserved DNA module array), spatial expression elements (e.g., differentiation of the palisade mesophyll cells, meristem expression, and endosperm expression), and biological process-related elements (including circadian control, cell cycle regulation, and light response) (Figure S6). The presence of these elements suggests that *DnaJ* genes were involved in both developmental processes and environmental stress responses in *G. hirsutum*.

### 2.5. Analysis of the DnaJs Expression Pattern

**Figure 4:**
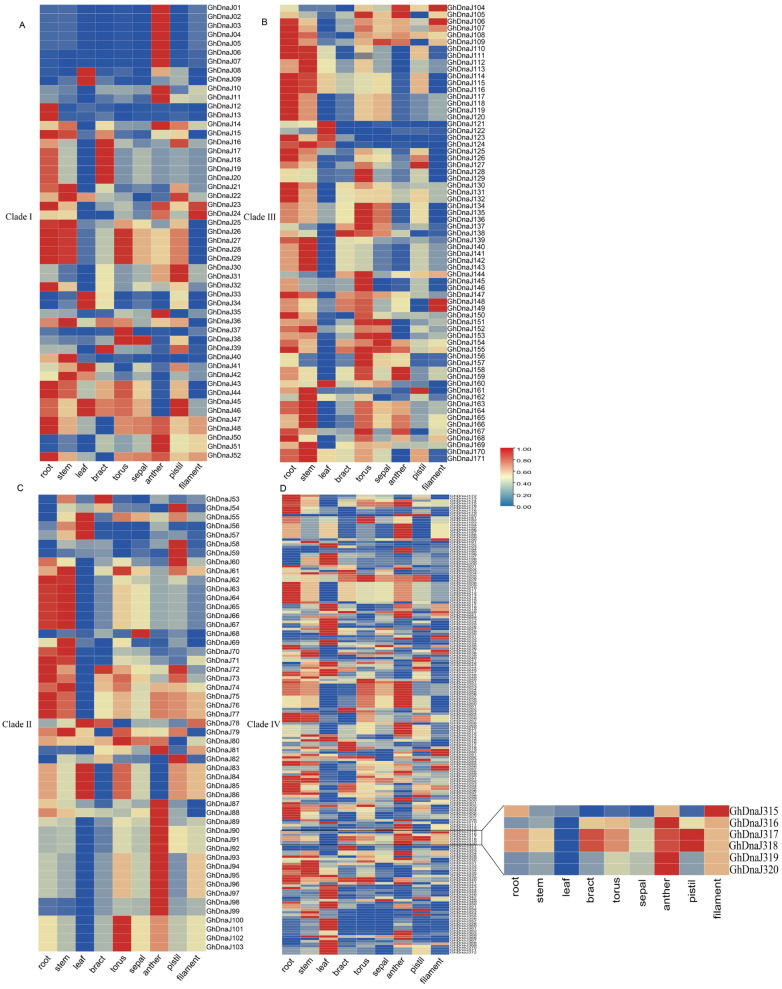
The expression pattern of the *DnaJ* gene family in different evolutionary branches in multiple tissues of upland cotton. (**A**) The expression pattern of *DnaJs* in Clade I. (**B**) The expression pattern of *DnaJs* in Clade II. (**C**) The expression pattern of *DnaJs* in Clade III. (**D**) The expression pattern of *DnaJs* in Clade IV.

Transcriptomic analysis via the CottonMD database (https://yanglab.hzau.edu.cn/CottonMD, accessed on 6 July 2025) revealed widespread expression of *DnaJ* homologs in upland cotton. Following exclusion of 13 genes with undetectable expression, the spatial expression profiles of 359 retained *DnaJ* genes were analyzed across multiple tissues, including root, stem, leaf, and reproductive tissues. The results demonstrated that genes such as *GhDnaJ124* (highly expressed in root) and *GhDnaJ21* (highly expressed in filament) were highly expressed in single tissues, suggesting their potential roles in specific developmental functions. *GhDnaJ341* exhibited high expression in stems, leaves, and receptacles, indicating its involvement in developmental regulation across multiple organs (Figure 4). *GhDnaJ190* showed notably high expression in bracts, while *GhDnaJ304* was highly expressed in receptacles, suggesting both may function as core regulatory genes. It is worth noting that *GhDnaJ316* displayed relatively high expression in both anthers and filaments, implying its critical role in floral development. It is critical to clarify that the term “floral development” in this context specifically denotes the maturation phase of floral organs subsequent to their initial formation, rather than the processes of organogenesis or primordial differentiation.

### 2.6. Functional Validation of GhDnaJ316 in the Floral Development of Upland Cotton

**Figure 5:**
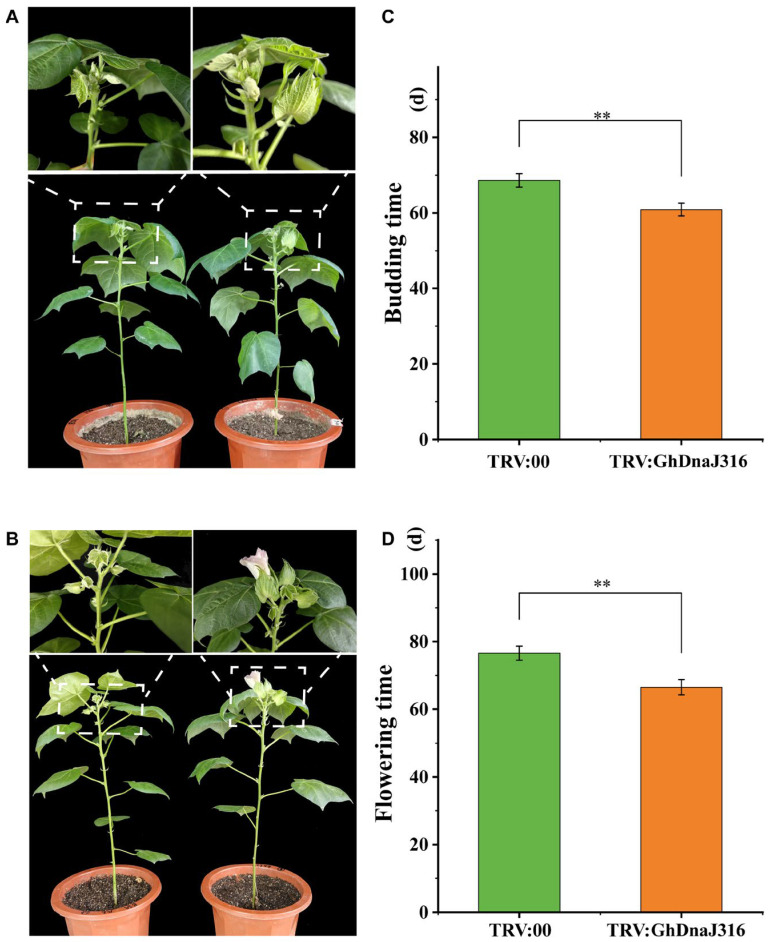
Silencing of *GhDnaJ316* promotes cotton budding and flowering time. (**A**) The phenotypes during budding time in control and *GhDnaJ316*-silenced plants. (**B**) The phenotypes for flowering time in control and *GhDnaJ316*-silenced plants. (**C**) The budding time statistics in control and *GhDnaJ316*-silenced plants. (**D**) The flowering time statistics in control and *GhDnaJ316*-silenced plants. The asterisks “**” here indicate that the significance level reached *p* < 0.01.

During floral organ development in *G*. *hirsutum*, stamens form first, followed by the differentiation of stamen primordia into anthers and filaments. Thus, to identify *DnaJ* family members associated with floral development, we prioritized *GhDnaJ* genes exhibiting high expression in anthers and filaments. Intriguingly, *GhDnaJ316* showed pronounced expression in these tissues, suggesting its likely association with floral development. Consequently, we chose *GhDnaJ316* to elucidate its function in regulating flowering time via virus-induced gene silencing assays (VIGS). The VIGS here was performed using a TRV-based vector to achieve rapid and efficient target gene suppression, enabling systematic statistics of phenotypic changes in *GhDnaJ316*-silenced plants (Figure 5A,B). qRT–PCR analysis confirmed significant knockdown of target gene expression in TRV:*GhDnaJ316* plants compared to empty vector controls, validating effective silencing (*p* < 0.05, Figure S7, Table S4). Compared to controls, TRV:*GhDnaJ316* plants exhibited precocious budding (7.7 days earlier, *p* < 0.01) and flowering (9.7 days earlier, *p* < 0.01) (Figure 5C,D, Tables S5 and S6), indicating that *GhDnaJ316* functions as a negative regulator of floral transition and development.

## 3. Discussion

As a crucial natural textile fiber and oilseed crop, cotton ranks among the highest-valued economic crops globally, serving as a primary source of edible oil and livestock feed derivatives [33]. However, cotton plants are frequently confronted with abiotic stresses such as drought, low accumulated temperature, and short frost-free periods [34,35]. These climatic constraints adversely affect plant growth and development, ultimately compromising cotton yield and fiber quality. The development and breeding of early-maturing cotton germplasm can effectively mitigate the impacts of such abiotic stresses. Therefore, identifying key genes associated with early-maturity traits such as early flowering, is crucial for enhancing yield and agronomic performance in cotton [36,37,38]. DnaJ cochaperones form dynamic complexes with Hsp70 chaperones to regulate ATP hydrolysis-driven conformational changes, thereby facilitating protein folding, substrate translocation, and degradation of misfolded polypeptides through allosteric modulation of the Hsp70 functional cycle [39,40]. Despite the established significance of the DnaJ-Hsp70 chaperone complex in proteostasis, the expression dynamics and functional diversification of the DnaJ protein family in cotton remain to be fully elucidated.

Systematic identification of gene families in genomes serves as a cornerstone for elucidating evolutionary relationship and inferring functional attributes of genes, thereby enabling the formulation and empirical validation of hypotheses regarding their biological roles [41,42,43]. Recent studies have identified DnaJ proteins as ubiquitously involved in diverse plant growth and developmental processes across several species, including pepper, soybean, and wheat [44,45,46]. In this study, a genome-wide investigation elucidated the phylogenetic relationships and genetic characteristics of entire 372 *DnaJ* genes in *G. hirsutum*, integrating phylogenetic reconstruction, conserved domain profiling, and structural diversification analyses to delineate evolutionary trajectories and functional divergence. We observed that the *DnaJ* gene family exhibits a distribution bias toward telomeric regions rather than centromeric regions across chromosomes. This distributional preference might be associated with the cotton-specific genomic architecture, where centromeric regions are predominantly characterized by high-density repetitive sequences and a paucity of functional genes [47]. The phylogenetic tree revealed that the *DnaJ* gene family diverged into four major evolutionary clades, with significant variations in genomic structure composition and conserved motifs among members of these clades. This observation aligns with the canonical classification of the DnaJ protein family into Type I to Type IV, which is defined by conserved structural domains [6,48]. A similar phylogenetic topology is exhibited in *Vitis vinifera* in terms of this gene family [49]. This pattern suggests that the *DnaJ* genes in upland cotton likely underwent horizontal gene duplication events, leading to the formation of distinct paralogous subfamilies. Genomic diversification concomitantly drives functional divergence within the gene family, as evidenced by differential expression patterns and stress-responsive specialization among subfamily members [50]. In essence, the retention and subsequent functional diversification of duplicated genes serve as an evolutionary substrate upon which natural selection acts, thereby facilitating the emergence of adaptive traits that confer competitive advantages in dynamic ecological contexts [51].

Cis-acting elements function as critical genomic determinants that orchestrate plant developmental programs and environmental adaptations through sequence-specific interactions with transcription factors. These interactions precisely modulate transcriptional initiation rates and efficiency by recruiting RNA polymerase complexes to core promoters, thereby establishing spatiotemporal control of gene expression networks [52]. Comprehensive cis-acting element profiling in the *GhDnaJ* gene family promoters identified diverse motifs, including circadian-associated elements, cell cycle-regulated elements, and photoresponsive motifs that coordinate developmental processes. This configuration suggests the hypothesis that *GhDnaJ* genes modulate biosynthetic and metabolic regulation during both vegetative and reproductive development in cotton [53]. Transcriptional profiling of *GhDnaJ* genes revealed elevated expression across multiple tissues, with *GhDnaJ316* exhibiting notably high transcript abundance in both anthers and filaments, suggesting its potential involvement in floral development (Figure 4). For functional validation, we performed silencing experiment on *GhDnaJ316* gene. The results revealed that the silencing of *GhDnaJ316* via VIGS accelerated floral transition in cotton, evidenced by precocious budding and flowering times compared to controls. These findings revealed that *GhDnaJ316* act a role as a negative regulator of floral development.

## 4. Materials and Methods

### 4.1. Genome-Wide Identification of DnaJ Gene Family

The whole-genome sequencing data of *Gossypium hirsutum* (upland cotton) were retrieved from the Phytozome database (https://phytozome-next.jgi.doe.gov/, accessed on 2 April 2025). The hidden Markov model (HMM) profile for DnaJ genes, downloaded from the Pfam database, was employed as an informational probe for domain retrieval using HMMER software(http://www.ebi.ac.uk/Tools/hmmer, accessed on 11 April 2025) [54]. Concurrently, DnaJ protein sequences of *Arabidopsis thaliana* were obtained from the TAIR database (https://www.arabidopsis.org/, accessed on 14 April 2025) and subjected to BLASTP analysis (E-value set to 1 × 10^−5^) to identify homologous sequences. The results from both HMMER (hmmsearch) and BLASTP analyses were consolidated, and redundant or incomplete sequences were removed. Conserved domains were subsequently validated using the NCBI Conserved Domain Database (CDD; https://www.ncbi.nlm.nih.gov/, accessed on 20 April 2025). Sequences lacking essential structural domains characteristic of the *DnaJ* gene family were excluded, yielding the final set of *DnaJ* gene family sequences.

### 4.2. Physicochemical Properties and Subcellular Localization

Physicochemical properties of DnaJ family members including amino acid counts, molecular weight, isoelectric point (pI), instability index, grand average of hydropathicity (GRAVY), and aliphatic index, were predicted using the online ProtParam tool (https://web.expasy.org/protparam/, accessed on 29 April 2025). The predictions for subcellular localization were performed using WoLF PSORT (https://wolfpsort.hgc.jp/, accessed on 8 May 2025) [55].

### 4.3. Chromosomal Localization and Structural Prediction

Chromosomal positions of *DnaJ* gene family were extracted from the annotated gff3 file. The TBtools v2.331 software [56] was used to map the distribution of *DnaJ* genes across chromosomes. Secondary and tertiary structures of DnaJ proteins were predicted using SOPMA (https://npsa-prabi.ibcp.fr/cgi-bin/npsa_automat.pl?page=/NPSA/npsa_sopma.html, accessed on 23 May 2025) [57] and SWISS-MODEL (https://swissmodel.expasy.org, accessed on 26 May 2025) [58].

### 4.4. Analysis of Conserved Motifs, Gene Structure, and Cis-Acting Elements

Conserved motifs within DnaJ protein sequences were identified using MEME (https://gensoft.pasteur.fr/docs/meme/4.11.2/meme-chip.html, accessed on 30 May 2025) with the multiple expectation maximization for motif elicitation algorithm, setting the maximum number of motifs to 15 [59]. Then, gene structures were predicted using GSDS v2.0 (http://GSDS.cbi.pku.edu.cn, accessed on 1 June 2025) [60]. To identify cis-elements, the promoter sequences including 2000 bp upstream of the start codon were analyzed using PlantCARE (http://bioinformatics.psb.ugent.be/webtools/plantcare/html/, accessed on 4 June 2025). And elements responsive to environmental stimuli, hormones, and developmental processes were statistically analyzed.

### 4.5. Phylogenetic Analysis of the DnaJ Gene Family

To investigate evolutionary relationships among *DnaJ* family members, the protein sequences from *G. hirsutum* were aligned using MAFFT [61], and a maximum-likelihood phylogenetic tree was constructed using IQ-TREE with 1000 bootstrap replicates. The tree was visualized via the iTOL web server (https://itol.embl.de/, accessed on 13 June 2025).

### 4.6. Tissue-Specific Expression Analysis

Transcriptome data from *G. hirsutum* tissues (root, stem, leaf, bract, torus, sepal, anther, pistil and filament) were analyzed to determine *DnaJ* gene expression patterns. TPM (transcripts per million) values for *DnaJ* genes were calculated using the RPKM/FPKM and TPM Calculator subroutine in TBtools. Log-transformed TPM values were used to generate expression heatmaps. Differential expression analysis across tissues was performed using Rstudio v4.3.2 [62].

### 4.7. Silencing of GhDnaJ316 by VIGS in Upland Cotton

Through transcriptome expression pattern analysis and literature review, we identified that *GhDnaJ316* in the *DnaJ* gene family may regulate flowering in *G*. *hirsutum*. To functionally validate this gene, we silenced *GhDnaJ316* using VIGS. Target-specific silenced fragments (300–500 bp) were cloned into the TRV-based vector pYL156, and the resulting constructs were transformed into *Agrobacterium* strain GV3101 using a freeze–thaw method [30]. Primers for constructing the TRV-based vector pYL156 to silence the target gene are listed in Table S1. Helper vector pYL192 was combined with TRV constructs (TRV:*GhDnaJ316* and TRV:00) at a 1:1 ratio, incubated at 28 °C for 3 h, and co-infiltrated into cotyledons of 8-day-old cotton plants. Virus-infected seedlings were incubated in darkness at 25 °C for 24 h, then transferred to a 16-h-light/8-h-dark photoperiod. Following albinism emergence in the positive control (TRV:*GhDnaJ316*), plants with gene expression levels below 50% of TRV:00 controls were selected by qRT-PCR and designated as TRV:*GhDnaJ316* plants. Here we implemented biological replicated pooling for both the experimental group (TRV:*GhDnaJ316*) and control group (TRV:00) to ensure data representativeness and minimize individual variation bias [63,64]. Finally, we quantified two phenological traits: budding time (defined as the first occurrence of floral buds ≥ 2 mm in diameter at the shoot apical meristem) and flowering time (the duration from budding to the full anthesis of the first flower). in the TRV:00 and TRV:*GhDnaJ316* plants. It is to be clarified that the observed sample size discrepancy arises from differential phenotyping feasibility across developmental stages, wherein rapid bud enclosure during squaring impedes precise trait capture, while stabilized floral phenotypes at flowering enable complete recording—a well-documented limitation in plant developmental biology due to trait observability constraints. Despite this variation, the effective sample size for phenological traits analysis meets field-accepted statistical standards [33].

## 5. Conclusions

In the present study, a total of 372 *DnaJ* genes were identified in *Gossypium hirsutum*, which were phylogenetically classified into four clades with clade-specific structural variations in intron architecture and conserved motifs. Chromosomal distribution analysis revealed non-uniform localization. Cis-element profiling implicated *GhDnaJs* in regulating biosynthetic and metabolic pathways during vegetative and reproductive development. Tissue-specific expression revealed the high expression of *GhDnaJ316* in anthers and filaments. VIGS-mediated silencing confirmed *GhDnaJ316* as a negative regulator of floral transition, accelerating budding and flowering times by 7.7 days and 9.7 days, respectively, in silenced plants. These findings elucidated the genomic features of *GhDnaJs* and its negative regulator role in floral development, laying a foundational framework for further elucidating their roles in cotton early-maturing trait development, providing valuable implications for cotton breeding programs.

## Data Availability

Data are contained within the article and Supplementary Materials. Transcriptome data for different tissues were obtained from public databases.

## Supplementary Materials

The following supporting information can be downloaded at https://www.mdpi.com/article/10.3390/plants14213380/s1: Figure S1: Subcellular localization of DnaJ protein members in *G. hirsutum*; Figure S2: Phylogenetic relationships of the DnaJ proteins in *G. hirsutum* and *A. thaliana*. (A) Circular phylogenetic tree. (B) Radial phylogenetic tree diagram; Figure S3: The conserved motifs of *GhDnaJ* genes; Figure S4: Secondary structure predictions of DnaJ protein members. Light green represents α-helices, yellow denotes β-sheets, and dark green indicates random coils; Figure S5: Tertiary structure of representative *DnaJ* family members from major clades in the phylogenetic tree; Figure S6: The prediction of cis-acting elements of *GhDnaJ* genes within individual phylogenetic clades; Figure S7: qRT–PCR analysis of *GhDnaJ316* expression in empty vector and silenced (TRV:*GhDnaJ316*) plants; Table S1: Fluorescent quantitative primers; Table S2: The location statistics of *DnaJ* gene family within individual phylogenetic clades; Table S3: Physicochemical properties of DnaJ protein members in *G. hirsutum*; Table S4: Statistics of relative expression in empty vector and silenced plants; Table S5: Statistics of budding time in empty vector and silenced plants; Table S6: Statistics of flowering time in empty vector and silenced plants; Data S1: The sequences of 372 DnaJ proteins within upland cotton.
