# Supplementary material for: Genome-Wide Identification of DnaJ Gene Family and VIGS Analysis Reveal the Function of GhDnaJ316 in Floral Development for Upland Cotton
Source: Plants (Basel). 2025 Nov 5;14(21):3380. doi: 10.3390/plants14213380 (PMC12609765; doi:10.3390/plants14213380)
Supplement: Supplementary file 1 [file plants-14-03380-s001.zip › Fig.S5.pdf]

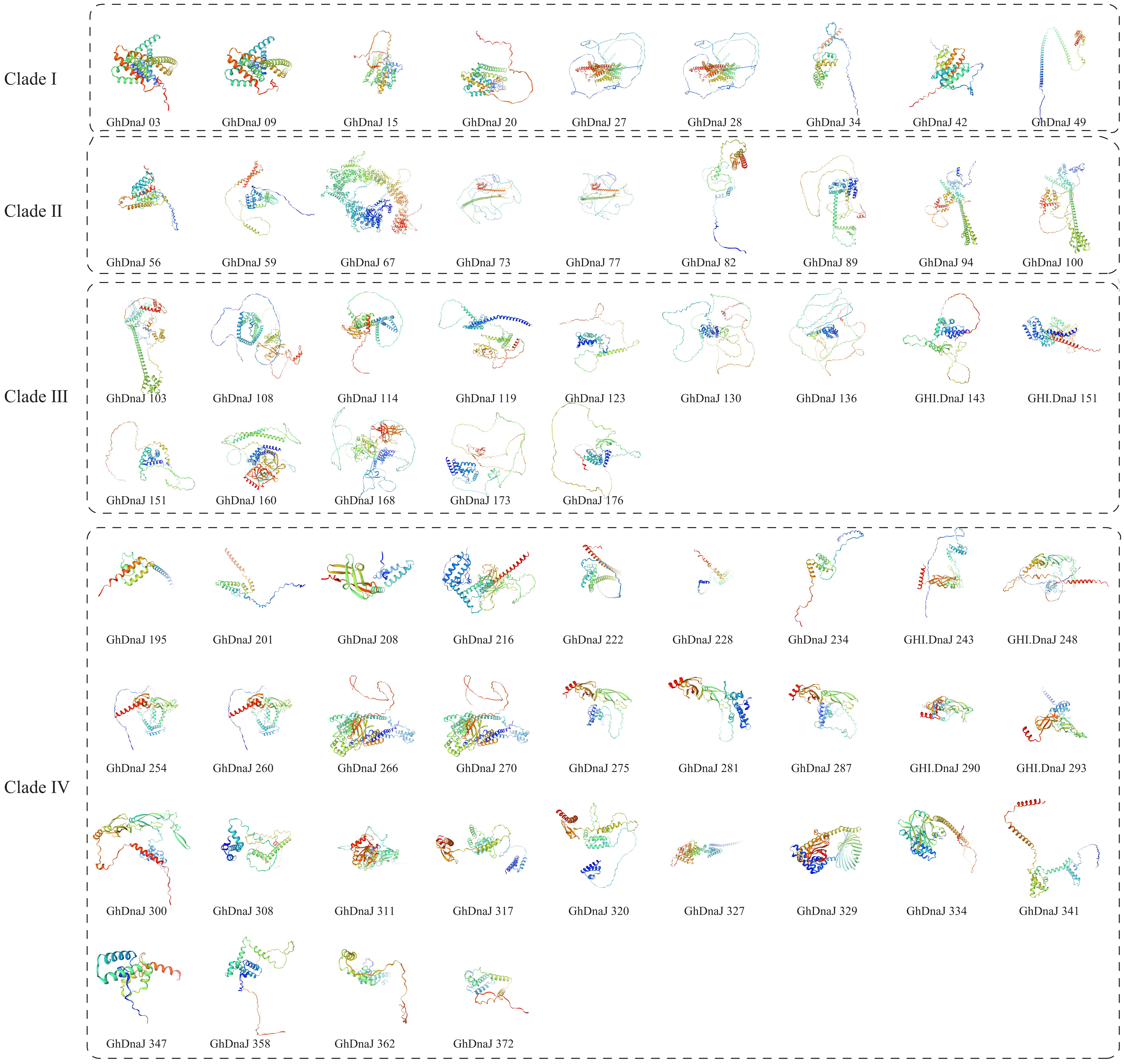

Clade I
GhDnaJ 03
GhDnaJ 09
GhDnaJ 15
GhDnaJ 20
GhDnaJ 27
GhDnaJ 28
GhDnaJ 34
GhDnaJ 42
GhDnaJ 49
Clade II
GhDnaJ 56
GhDnaJ 59
GhDnaJ 67
GhDnaJ 73
GhDnaJ 77
GhDnaJ 82
GhDnaJ 89
GhDnaJ 94
GhDnaJ 100
Clade III
GhDnaJ 103
GhDnaJ 108
GhDnaJ 114
GhDnaJ 119
GhDnaJ 123
GhDnaJ 130
GhDnaJ 136
GHI.DnaJ 143
GHI.DnaJ 151
GhDnaJ 151
GhDnaJ 160
GhDnaJ 168
GhDnaJ 173
GhDnaJ 176
Clade IV
GhDnaJ 195
GhDnaJ 201
GhDnaJ 208
GhDnaJ 216
GhDnaJ 222
GhDnaJ 228
GhDnaJ 234
GHI.DnaJ 243
GHI.DnaJ 248
GhDnaJ 254
GhDnaJ 260
GhDnaJ 266
GhDnaJ 270
GhDnaJ 275
GhDnaJ 281
GhDnaJ 287
GHI.DnaJ 290
GHI.DnaJ 293
GhDnaJ 300
GhDnaJ 308
GhDnaJ 311
GhDnaJ 317
GhDnaJ 320
GhDnaJ 327
GhDnaJ 329
GhDnaJ 334
GhDnaJ 341
GhDnaJ 347
GhDnaJ 358
GhDnaJ 362
GhDnaJ 372
