# Supplementary material for: Genome-Wide Identification of DnaJ Gene Family and VIGS Analysis Reveal the Function of GhDnaJ316 in Floral Development for Upland Cotton
Source: Plants (Basel). 2025 Nov 5;14(21):3380. doi: 10.3390/plants14213380 (PMC12609765; doi:10.3390/plants14213380)
Supplement: Supplementary file 1 [file plants-14-03380-s001.zip › Fig.S6.pdf]

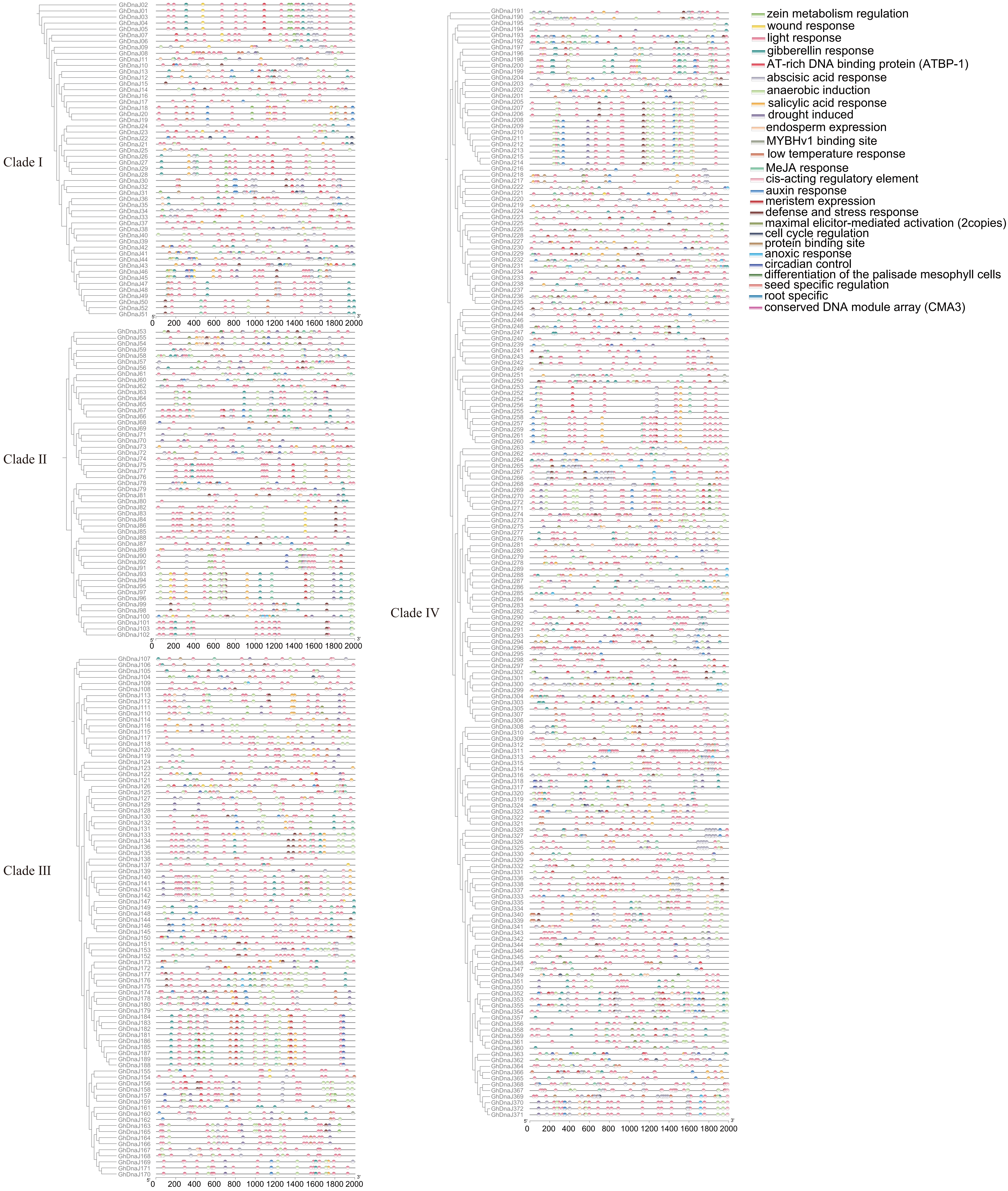
Clade I
Clade II
Clade III
Clade IV
5'
0
200
400
600
800
1000
1200
1400
1600
1800
2000
3'
zein metabolism regulation
wound response
light response
gibberellin response
AT-rich DNA binding protein (ATBP-1)
abscisic acid response
anaerobic induction
salicylic acid response
drought induced
endosperm expression
MYBHv1 binding site
low temperature response
MeJA response
cis-acting regulatory element
auxin response
meristem expression
defense and stress response
maximal elicitor-mediated activation (2copies)
cell cycle regulation
protein binding site
anoxic response
circadian control
differentiation of the palisade mesophyll cells
seed specific regulation
root specific
conserved DNA module array (CMA3)
