# Supplementary material for: Genome-Wide Identification of DnaJ Gene Family and VIGS Analysis Reveal the Function of GhDnaJ316 in Floral Development for Upland Cotton
Source: Plants (Basel). 2025 Nov 5;14(21):3380. doi: 10.3390/plants14213380 (PMC12609765; doi:10.3390/plants14213380)
Supplement: Supplementary file 1 [file plants-14-03380-s001.zip › Table S1.pdf]

Table S1 Fluorescent quantitative primers.

**Primer**	**Sequence (5'-3')**
GhDnaJ316-F	TAAGACTGTGTGCAAGGCGT
GhDnaJ316-R	TTGGCTCTAGCAGCACTTCC
