# Supplementary material for: Genome-Wide Identification of DnaJ Gene Family and VIGS Analysis Reveal the Function of GhDnaJ316 in Floral Development for Upland Cotton
Source: Plants (Basel). 2025 Nov 5;14(21):3380. doi: 10.3390/plants14213380 (PMC12609765; doi:10.3390/plants14213380)
Supplement: Supplementary file 1 [file plants-14-03380-s001.zip › Table S2.pdf]

Table S2 The location statistics of *DnaJ* gene family in *Gossypium hirsutum*.

**Gene name**	**Gene id**	**Chromosome location**
GhDnaJ01	Gohir.A01G010300.1.v3.1	ChrA01:949492-952561
GhDnaJ02	Gohir.A01G010700.2.v3.1	ChrA01:963483-967091
GhDnaJ03	Gohir.A01G012600.1.v3.1	ChrA01:1316491-1319558
GhDnaJ04	Gohir.A01G019700.1.v3.1	ChrA01:1867803-1870052
GhDnaJ05	Gohir.A01G033400.1.v3.1	ChrA01:2675049-2676912
GhDnaJ06	Gohir.A01G036900.1.v3.1	ChrA01:2943376-2948916
GhDnaJ07	Gohir.A01G036900.2.v3.1	ChrA01:2943376-2948916
GhDnaJ08	Gohir.A01G110900.1.v3.1	ChrA01:21741185-21741685
GhDnaJ09	Gohir.A01G128100.1.v3.1	ChrA01:38726072-38727817
GhDnaJ10	Gohir.A01G135100.1.v3.1	ChrA01:48321371-48324554
GhDnaJ11	Gohir.A01G170400.1.v3.1	ChrA01:106805430-106808940
GhDnaJ12	Gohir.A01G187200.1.v3.1	ChrA01:111454059-111457583
GhDnaJ13	Gohir.A01G195500.1.v3.1	ChrA01:113655935-113658419
GhDnaJ14	Gohir.A01G215700.1.v3.1	ChrA01:116952294-116956813
GhDnaJ15	Gohir.A01G216700.1.v3.1	ChrA01:117064991-117066946
GhDnaJ16	Gohir.A01G227100.1.v3.1	ChrA01:118241074-118244268
GhDnaJ17	Gohir.A02G019600.1.v3.1	ChrA02:2310548-2313087
GhDnaJ18	Gohir.A02G045600.1.v3.1	ChrA02:6302039-6304837
GhDnaJ19	Gohir.A02G045600.2.v3.1	ChrA02:6301702-6305907
GhDnaJ20	Gohir.A02G049800.1.v3.1	ChrA02:7144010-7146623
GhDnaJ21	Gohir.A02G077500.1.v3.1	ChrA02:17186528-17188989
GhDnaJ22	Gohir.A02G077500.2.v3.1	ChrA02:17186566-17188966

GhDnaJ23	Gohir.A02G078800.1.v3.1	ChrA02:18514499-18521066
GhDnaJ24	Gohir.A02G107229.1.v3.1	ChrA02:74361762-74366903
GhDnaJ25	Gohir.A02G109400.1.v3.1	ChrA02:67442442-67446542
GhDnaJ26	Gohir.A02G126800.1.v3.1	ChrA02:93662737-93667673
GhDnaJ27	Gohir.A02G126800.2.v3.1	ChrA02:93662891-93667639
GhDnaJ28	Gohir.A02G132400.1.v3.1	ChrA02:97480142-97480639
GhDnaJ29	Gohir.A02G142300.2.v3.1	ChrA02:101550573-101555238
GhDnaJ30	Gohir.A02G153400.1.v3.1	ChrA02:103737442-103741938
GhDnaJ31	Gohir.A02G153400.3.v3.1	ChrA02:103737271-103741803
GhDnaJ32	Gohir.A02G171000.1.v3.1	ChrA02:105953128-105954308
GhDnaJ33	Gohir.A03G008300.1.v3.1	ChrA03:1695136-1700265
GhDnaJ34	Gohir.A03G064500.1.v3.1	ChrA03:16809061-16812745
GhDnaJ35	Gohir.A03G067500.1.v3.1	ChrA03:18279969-18292736
GhDnaJ36	Gohir.A03G067500.2.v3.1	ChrA03:18279969-18292736
GhDnaJ37	Gohir.A03G078312.1.v3.1	ChrA03:33347331-33349735
GhDnaJ38	Gohir.A03G078312.2.v3.1	ChrA03:33346968-33349735
GhDnaJ39	Gohir.A03G078312.3.v3.1	ChrA03:33347455-33349735
GhDnaJ40	Gohir.A03G078312.4.v3.1	ChrA03:33346967-33349735
GhDnaJ41	Gohir.A03G078312.5.v3.1	ChrA03:33346967-33349735
GhDnaJ42	Gohir.A03G078312.6.v3.1	ChrA03:33346966-33349735
GhDnaJ43	Gohir.A03G078312.7.v3.1	ChrA03:33347019-33349735
GhDnaJ44	Gohir.A03G078312.8.v3.1	ChrA03:33346968-33349735
GhDnaJ45	Gohir.A03G092100.1.v3.1	ChrA03:45163377-45175110
GhDnaJ46	Gohir.A03G122000.2.v3.1	ChrA03:87698880-87700019
GhDnaJ47	Gohir.A03G125200.1.v3.1	ChrA03:88822297-88826185
GhDnaJ48	Gohir.A03G125200.2.v3.1	ChrA03:88822297-88826185

GhDnaJ49	Gohir.A03G142900.1.v3.1	ChrA03:98821009-98828220
GhDnaJ50	Gohir.A03G142900.3.v3.1	ChrA03:98821340-98828220
GhDnaJ51	Gohir.A03G145800.1.v3.1	ChrA03:99571140-99573851
GhDnaJ52	Gohir.A03G204000.1.v3.1	ChrA03:110211495-110212168
GhDnaJ53	Gohir.A04G002500.1.v3.1	ChrA04:244563-245354
GhDnaJ54	Gohir.A04G065342.1.v3.1	ChrA04:55470633-55476568
GhDnaJ55	Gohir.A04G090400.5.v3.1	ChrA04:76547559-76550943
GhDnaJ56	Gohir.A04G093600.1.v3.1	ChrA04:77569057-77571987
GhDnaJ57	Gohir.A04G098700.1.v3.1	ChrA04:79557435-79563155
GhDnaJ58	Gohir.A04G115200.2.v3.1	ChrA04:84071305-84074441
GhDnaJ59	Gohir.A04G124400.1.v3.1	ChrA04:85646425-85650514
GhDnaJ60	Gohir.A04G124400.2.v3.1	ChrA04:85645988-85650598
GhDnaJ61	Gohir.A04G124400.4.v3.1	ChrA04:85646456-85650598
GhDnaJ62	Gohir.A04G124400.6.v3.1	ChrA04:85645929-85650514
GhDnaJ63	Gohir.A04G124400.7.v3.1	ChrA04:85645929-85650514
GhDnaJ64	Gohir.A05G010500.2.v3.1	ChrA05:1237845-1241258
GhDnaJ65	Gohir.A05G010500.3.v3.1	ChrA05:1237861-1241258
GhDnaJ66	Gohir.A05G017000.1.v3.1	ChrA05:1751223-1751870
GhDnaJ67	Gohir.A05G021000.1.v3.1	ChrA05:2104712-2118433
GhDnaJ68	Gohir.A05G039100.1.v3.1	ChrA05:3880709-3882108
GhDnaJ69	Gohir.A05G070300.1.v3.1	ChrA05:6710710-6718058
GhDnaJ70	Gohir.A05G077850.1.v3.1	ChrA05:7376315-7380995
GhDnaJ71	Gohir.A05G118300.1.v3.1	ChrA05:11207826-11209074
GhDnaJ72	Gohir.A05G138100.1.v3.1	ChrA05:13323744-13326166
GhDnaJ73	Gohir.A05G138100.2.v3.1	ChrA05:13323744-13326166
GhDnaJ74	Gohir.A05G158900.1.v3.1	ChrA05:15556937-15559398
GhDnaJ75	Gohir.A05G212600.1.v3.1	ChrA05:21114614-21118232
GhDnaJ76	Gohir.A05G212600.2.v3.1	ChrA05:21114962-21118313

GhDnaJ77	Gohir.A05G219800.2.v3.1	ChrA05:21929627-21931343
GhDnaJ78	Gohir.A05G286400.1.v3.1	ChrA05:35060946-35063982
GhDnaJ79	Gohir.A05G306500.1.v3.1	ChrA05:45073556-45075455
GhDnaJ80	Gohir.A05G306900.2.v3.1	ChrA05:45322551-45325409
GhDnaJ81	Gohir.A05G321800.1.v3.1	ChrA05:70658580-70666655
GhDnaJ82	Gohir.A05G344900.1.v3.1	ChrA05:86861323-86864337
GhDnaJ83	Gohir.A05G413500.1.v3.1	ChrA05:110633832-110639227
GhDnaJ84	Gohir.A06G008800.3.v3.1	ChrA06:815218-821249
GhDnaJ85	Gohir.A06G026800.1.v3.1	ChrA06:2936044-2939862
GhDnaJ86	Gohir.A06G026800.2.v3.1	ChrA06:2936044-2939862
GhDnaJ87	Gohir.A06G026800.3.v3.1	ChrA06:2936044-2939862
GhDnaJ88	Gohir.A06G032604.1.v3.1	ChrA06:4010517-4012060
GhDnaJ89	Gohir.A06G032676.1.v3.1	ChrA06:4253684-4260367
GhDnaJ90	Gohir.A06G032676.4.v3.1	ChrA06:4253684-4260746
GhDnaJ91	Gohir.A06G032676.5.v3.1	ChrA06:4253684-4260746
GhDnaJ92	Gohir.A06G032676.6.v3.1	ChrA06:4253684-4260367
GhDnaJ93	Gohir.A06G032676.7.v3.1	ChrA06:4253684-4260553
GhDnaJ94	Gohir.A06G047700.1.v3.1	ChrA06:8075007-8078397
GhDnaJ95	Gohir.A06G060200.2.v3.1	ChrA06:11978274-11990834
GhDnaJ96	Gohir.A06G060200.3.v3.1	ChrA06:11978274-11990834
GhDnaJ97	Gohir.A06G060200.4.v3.1	ChrA06:11977842-11987031
GhDnaJ98	Gohir.A06G060200.5.v3.1	ChrA06:11977842-11987031
GhDnaJ99	Gohir.A06G060200.6.v3.1	ChrA06:11977842-11987031
GhDnaJ100	Gohir.A06G113200.2.v3.1	ChrA06:55302333-55311258
GhDnaJ101	Gohir.A06G170000.1.v3.1	ChrA06:121750763-121753517
GhDnaJ102	Gohir.A06G172300.3.v3.1	ChrA06:122148509-122151692
GhDnaJ103	Gohir.A06G172300.4.v3.1	ChrA06:122148509-

		122151692
GhDnaJ104	Gohir.A06G172500.1.v3.1	ChrA06:122163829-122168715
GhDnaJ105	Gohir.A06G172500.2.v3.1	ChrA06:122163854-122168849
GhDnaJ106	Gohir.A06G172500.3.v3.1	ChrA06:122163820-122168849
GhDnaJ107	Gohir.A06G172500.4.v3.1	ChrA06:122163854-122168849
GhDnaJ108	Gohir.A06G172500.5.v3.1	ChrA06:122163854-122168849
GhDnaJ109	Gohir.A06G180700.1.v3.1	ChrA06:123478763-123487406
GhDnaJ110	Gohir.A07G050700.2.v3.1	ChrA07:6622998-6625271
GhDnaJ111	Gohir.A07G087500.1.v3.1	ChrA07:14867935-14870973
GhDnaJ112	Gohir.A07G113600.1.v3.1	ChrA07:21608361-21610900
GhDnaJ113	Gohir.A07G127700.1.v3.1	ChrA07:26853448-26856022
GhDnaJ114	Gohir.A07G131300.1.v3.1	ChrA07:27938574-27946954
GhDnaJ115	Gohir.A07G131300.3.v3.1	ChrA07:27938962-27946954
GhDnaJ116	Gohir.A07G164100.1.v3.1	ChrA07:74748520-74752081
GhDnaJ117	Gohir.A07G164100.2.v3.1	ChrA07:74748348-74752171
GhDnaJ118	Gohir.A07G194800.1.v3.1	ChrA07:91351194-91357744
GhDnaJ119	Gohir.A08G001000.1.v3.1	ChrA08:411961-414309
GhDnaJ120	Gohir.A08G001000.2.v3.1	ChrA08:411961-414309
GhDnaJ121	Gohir.A08G001000.3.v3.1	ChrA08:411961-414552
GhDnaJ122	Gohir.A08G053500.1.v3.1	ChrA08:7666587-7673020
GhDnaJ123	Gohir.A08G118800.1.v3.1	ChrA08:93506534-93510364
GhDnaJ124	Gohir.A08G153200.3.v3.1	ChrA08:109834031-109837104
GhDnaJ125	Gohir.A08G207100.4.v3.1	ChrA08:120623888-120626682

GhDnaJ126	Gohir.A08G207100.5.v3.1	ChrA08:120624113-120626675
GhDnaJ127	Gohir.A08G207100.6.v3.1	ChrA08:120623890-120626714
GhDnaJ128	Gohir.A08G221650.1.v3.1	ChrA08:122611684-122614315
GhDnaJ129	Gohir.A08G229700.1.v3.1	ChrA08:123639604-123642883
GhDnaJ130	Gohir.A09G028400.1.v3.1	ChrA09:9160795-9163353
GhDnaJ131	Gohir.A09G063400.1.v3.1	ChrA09:55889226-55892546
GhDnaJ132	Gohir.A09G100600.1.v3.1	ChrA09:66041139-66044672
GhDnaJ133	Gohir.A09G120600.1.v3.1	ChrA09:69638084-69650345
GhDnaJ134	Gohir.A09G120600.3.v3.1	ChrA09:69638084-69650345
GhDnaJ135	Gohir.A09G120600.4.v3.1	ChrA09:69638084-69650345
GhDnaJ136	Gohir.A09G120600.6.v3.1	ChrA09:69637957-69649956
GhDnaJ137	Gohir.A09G128100.1.v3.1	ChrA09:70738879-70744421
GhDnaJ138	Gohir.A09G128100.2.v3.1	ChrA09:70737653-70744421
GhDnaJ139	Gohir.A09G128100.3.v3.1	ChrA09:70738869-70744421
GhDnaJ140	Gohir.A09G128100.4.v3.1	ChrA09:70738878-70744421
GhDnaJ141	Gohir.A09G150600.1.v3.1	ChrA09:74404131-74408142
GhDnaJ142	Gohir.A09G166500.1.v3.1	ChrA09:75862854-75868076
GhDnaJ143	Gohir.A09G184100.1.v3.1	ChrA09:77849369-77854572
GhDnaJ144	Gohir.A10G015300.1.v3.1	ChrA10:1291336-1294391
GhDnaJ145	Gohir.A10G051800.1.v3.1	ChrA10:5417420-5419982
GhDnaJ146	Gohir.A10G051800.2.v3.1	ChrA10:5417420-5419982
GhDnaJ147	Gohir.A10G051800.4.v3.1	ChrA10:5417420-5419982
GhDnaJ148	Gohir.A10G051800.5.v3.1	ChrA10:5417420-5419982
GhDnaJ149	Gohir.A10G101935.1.v3.1	ChrA10:22634956-22639437
GhDnaJ150	Gohir.A10G174700.1.v3.1	ChrA10:100558311-100563179

GhDnaJ151	Gohir.A11G041900.1.v3.1	ChrA11:4055357-4056535
GhDnaJ152	Gohir.A11G053000.1.v3.1	ChrA11:5213454-5214375
GhDnaJ153	Gohir.A11G076900.1.v3.1	ChrA11:7379982-7383655
GhDnaJ154	Gohir.A11G110100.1.v3.1	ChrA11:11764031-11767032
GhDnaJ155	Gohir.A11G110500.1.v3.1	ChrA11:11861364-11865988
GhDnaJ156	Gohir.A11G110600.1.v3.1	ChrA11:11873635-11878703
GhDnaJ157	Gohir.A11G145800.1.v3.1	ChrA11:17729773-17738099
GhDnaJ158	Gohir.A11G174800.1.v3.1	ChrA11:24575463-24576901
GhDnaJ159	Gohir.A11G221275.1.v3.1	ChrA11:69656256-69660398
GhDnaJ160	Gohir.A11G228400.1.v3.1	ChrA11:74757713-74762421
GhDnaJ161	Gohir.A11G236800.1.v3.1	ChrA11:97068994-97071605
GhDnaJ162	Gohir.A11G236800.2.v3.1	ChrA11:97069009-97071356
GhDnaJ163	Gohir.A11G236800.3.v3.1	ChrA11:97068994-97071605
GhDnaJ164	Gohir.A11G265100.1.v3.1	ChrA11:110054389-110056894
GhDnaJ165	Gohir.A11G271400.1.v3.1	ChrA11:111378256-111380662
GhDnaJ166	Gohir.A12G015400.1.v3.1	ChrA12:2027774-2031236
GhDnaJ167	Gohir.A12G042600.1.v3.1	ChrA12:7760675-7764435
GhDnaJ168	Gohir.A12G079800.1.v3.1	ChrA12:68522784-68524052
GhDnaJ169	Gohir.A12G091600.1.v3.1	ChrA12:52870335-52878093
GhDnaJ170	Gohir.A12G126100.1.v3.1	ChrA12:84228259-84230473
GhDnaJ171	Gohir.A12G128200.1.v3.1	ChrA12:84528885-84530178
GhDnaJ172	Gohir.A12G182366.1.v3.1	ChrA12:97951257-97954005
GhDnaJ173	Gohir.A12G190100.1.v3.1	ChrA12:98871839-98874170
GhDnaJ174	Gohir.A12G253400.1.v3.1	ChrA12:106150875-106156484
GhDnaJ175	Gohir.A12G253400.3.v3.1	ChrA12:106150883-106156484
GhDnaJ176	Gohir.A12G253400.4.v3.1	ChrA12:106150850-106156480

GhDnaJ177	Gohir.A12G253400.5.v3.1	ChrA12:106151112-106156475
GhDnaJ178	Gohir.A13G009100.1.v3.1	ChrA13:976702-982797
GhDnaJ179	Gohir.A13G032700.1.v3.1	ChrA13:3878550-3881830
GhDnaJ180	Gohir.A13G051300.3.v3.1	ChrA13:7123086-7127232
GhDnaJ181	Gohir.A13G121400.1.v3.1	ChrA13:86622627-86629232
GhDnaJ182	Gohir.A13G130114.1.v3.1	ChrA13:91119197-91120067
GhDnaJ183	Gohir.A13G153500.5.v3.1	ChrA13:98639412-98649901
GhDnaJ184	Gohir.A13G153500.7.v3.1	ChrA13:98639684-98649427
GhDnaJ185	Gohir.A13G153500.8.v3.1	ChrA13:98639412-98649901
GhDnaJ186	Gohir.A13G153500.9.v3.1	ChrA13:98639412-98649901
GhDnaJ187	Gohir.A13G199500.1.v3.1	ChrA13:107409652-107412766
GhDnaJ188	Gohir.A13G199500.2.v3.1	ChrA13:107409652-107412765
GhDnaJ189	Gohir.A13G217200.1.v3.1	ChrA13:109789440-109797082
GhDnaJ190	Gohir.D01G009900.1.v3.1	ChrD01:822856-828343
GhDnaJ191	Gohir.D01G010300.2.v3.1	ChrD01:836861-839904
GhDnaJ192	Gohir.D01G010300.3.v3.1	ChrD01:836855-840100
GhDnaJ193	Gohir.D01G013800.1.v3.1	ChrD01:1068599-1071683
GhDnaJ194	Gohir.D01G018300.1.v3.1	ChrD01:1556340-1558631
GhDnaJ195	Gohir.D01G023508.1.v3.1	ChrD01:2320903-2323007
GhDnaJ196	Gohir.D01G024200.1.v3.1	ChrD01:2625919-2631221
GhDnaJ197	Gohir.D01G024200.2.v3.1	ChrD01:2625919-2631221
GhDnaJ198	Gohir.D01G097600.1.v3.1	ChrD01:17173951-17175130
GhDnaJ199	Gohir.D01G116600.1.v3.1	ChrD01:25452533-25454295
GhDnaJ200	Gohir.D01G116600.2.v3.1	ChrD01:25452535-25454240
GhDnaJ201	Gohir.D01G122300.1.v3.1	ChrD01:29184232-29187470
GhDnaJ202	Gohir.D01G122300.2.v3.1	ChrD01:29184232-29187478

GhDnaJ203	Gohir.D01G161400.1.v3.1	ChrD01:56404774-56407667
GhDnaJ204	Gohir.D01G182500.1.v3.1	ChrD01:60742078-60745419
GhDnaJ205	Gohir.D01G185900.1.v3.1	ChrD01:61436044-61438575
GhDnaJ206	Gohir.D01G204500.1.v3.1	ChrD01:64047016-64051622
GhDnaJ207	Gohir.D01G205500.1.v3.1	ChrD01:64125765-64127551
GhDnaJ208	Gohir.D01G216900.1.v3.1	ChrD01:65233551-65236765
GhDnaJ209	Gohir.D02G022900.1.v3.1	ChrD02:2855030-2857539
GhDnaJ210	Gohir.D02G051600.1.v3.1	ChrD02:6521999-6526336
GhDnaJ211	Gohir.D02G054900.1.v3.1	ChrD02:7216282-7218087
GhDnaJ212	Gohir.D02G054900.2.v3.1	ChrD02:7216083-7218087
GhDnaJ213	Gohir.D02G084900.1.v3.1	ChrD02:15022462-15024832
GhDnaJ214	Gohir.D02G086000.3.v3.1	ChrD02:15893158-15895139
GhDnaJ215	Gohir.D02G086000.4.v3.1	ChrD02:15893158-15895139
GhDnaJ216	Gohir.D02G086000.5.v3.1	ChrD02:15893158-15895139
GhDnaJ217	Gohir.D02G086000.6.v3.1	ChrD02:15893158-15895139
GhDnaJ218	Gohir.D02G092000.1.v3.1	ChrD02:19487377-19489546
GhDnaJ219	Gohir.D02G092000.2.v3.1	ChrD02:19486829-19489546
GhDnaJ220	Gohir.D02G092000.3.v3.1	ChrD02:19486829-19489546
GhDnaJ221	Gohir.D02G120900.1.v3.1	ChrD02:42161704-42173175
GhDnaJ222	Gohir.D02G120900.3.v3.1	ChrD02:42161703-42173176
GhDnaJ223	Gohir.D02G144900.1.v3.1	ChrD02:54716957-54719060
GhDnaJ224	Gohir.D02G147933.1.v3.1	ChrD02:56546377-56550592
GhDnaJ225	Gohir.D02G147933.2.v3.1	ChrD02:56546376-56550592
GhDnaJ226	Gohir.D02G166200.1.v3.1	ChrD02:62389262-62396530
GhDnaJ227	Gohir.D02G169200.1.v3.1	ChrD02:62959496-62962153
GhDnaJ228	Gohir.D02G225300.1.v3.1	ChrD02:71022323-71022655
GhDnaJ229	Gohir.D03G009100.1.v3.1	ChrD03:611847-613154
GhDnaJ230	Gohir.D03G027000.1.v3.1	ChrD03:2691134-2695565
GhDnaJ231	Gohir.D03G038000.1.v3.1	ChrD03:4501279-4505949

GhDnaJ232	Gohir.D03G043800.1.v3.1	ChrD03:5999397-6000075
GhDnaJ233	Gohir.D03G054300.1.v3.1	ChrD03:9549790-9554845
GhDnaJ234	Gohir.D03G062200.1.v3.1	ChrD03:18701814-18706043
GhDnaJ235	Gohir.D03G070900.1.v3.1	ChrD03:28351389-28356146
GhDnaJ236	Gohir.D03G070900.2.v3.1	ChrD03:28351389-28356580
GhDnaJ237	Gohir.D03G090800.1.v3.1	ChrD03:37136372-37140121
GhDnaJ238	Gohir.D03G094000.1.v3.1	ChrD03:38133172-38146009
GhDnaJ239	Gohir.D03G162600.1.v3.1	ChrD03:52689811-52695075
GhDnaJ240	Gohir.D04G002600.1.v3.1	ChrD04:601474-606948
GhDnaJ241	Gohir.D04G002600.2.v3.1	ChrD04:601099-606948
GhDnaJ242	Gohir.D04G083300.1.v3.1	ChrD04:18260535-18263498
GhDnaJ243	Gohir.D04G102900.1.v3.1	ChrD04:37023254-37029025
GhDnaJ244	Gohir.D04G102900.2.v3.1	ChrD04:37023273-37029025
GhDnaJ245	Gohir.D04G102900.3.v3.1	ChrD04:37023319-37029030
GhDnaJ246	Gohir.D04G130500.1.v3.1	ChrD04:47904636-47907328
GhDnaJ247	Gohir.D04G132900.2.v3.1	ChrD04:48591752-48598631
GhDnaJ248	Gohir.D04G132900.3.v3.1	ChrD04:48591528-48593174
GhDnaJ249	Gohir.D04G138300.1.v3.1	ChrD04:50008238-50014299
GhDnaJ250	Gohir.D04G138300.2.v3.1	ChrD04:50008238-50014299
GhDnaJ251	Gohir.D04G156700.1.v3.1	ChrD04:53520208-53523496
GhDnaJ252	Gohir.D04G156700.2.v3.1	ChrD04:53519804-53523496
GhDnaJ253	Gohir.D04G156700.3.v3.1	ChrD04:53519916-53523496
GhDnaJ254	Gohir.D04G166500.2.v3.1	ChrD04:54764950-54769543
GhDnaJ255	Gohir.D04G166500.3.v3.1	ChrD04:54765465-54769543
GhDnaJ256	Gohir.D05G011300.1.v3.1	ChrD05:1107886-1111185
GhDnaJ257	Gohir.D05G017900.1.v3.1	ChrD05:1627591-1628238
GhDnaJ258	Gohir.D05G022400.2.v3.1	ChrD05:1963789-1974203
GhDnaJ259	Gohir.D05G040600.1.v3.1	ChrD05:3323919-3325291
GhDnaJ260	Gohir.D05G073300.1.v3.1	ChrD05:5889779-5897244

GhDnaJ261	Gohir.D05G080650.1.v3.1	ChrD05:6578511-6583220
GhDnaJ262	Gohir.D05G119000.1.v3.1	ChrD05:10036060-10037271
GhDnaJ263	Gohir.D05G140200.1.v3.1	ChrD05:12003024-12005246
GhDnaJ264	Gohir.D05G140200.3.v3.1	ChrD05:12003024-12005246
GhDnaJ265	Gohir.D05G161600.1.v3.1	ChrD05:14114441-14116851
GhDnaJ266	Gohir.D05G215800.1.v3.1	ChrD05:18959409-18963020
GhDnaJ267	Gohir.D05G215800.5.v3.1	ChrD05:18959235-18963149
GhDnaJ268	Gohir.D05G222400.3.v3.1	ChrD05:19778291-19779947
GhDnaJ269	Gohir.D05G222400.4.v3.1	ChrD05:19778291-19779947
GhDnaJ270	Gohir.D05G287500.1.v3.1	ChrD05:29362415-29365546
GhDnaJ271	Gohir.D05G287500.2.v3.1	ChrD05:29361564-29365546
GhDnaJ272	Gohir.D05G306500.1.v3.1	ChrD05:35262732-35264656
GhDnaJ273	Gohir.D05G307200.1.v3.1	ChrD05:35407677-35412061
GhDnaJ274	Gohir.D05G324100.1.v3.1	ChrD05:49583922-49591817
GhDnaJ275	Gohir.D05G386900.1.v3.1	ChrD05:65946559-65947564
GhDnaJ276	Gohir.D06G004400.3.v3.1	ChrD06:668522-674564
GhDnaJ277	Gohir.D06G024100.1.v3.1	ChrD06:2622883-2626812
GhDnaJ278	Gohir.D06G024100.2.v3.1	ChrD06:2622883-2626812
GhDnaJ279	Gohir.D06G024100.4.v3.1	ChrD06:2622841-2626760
GhDnaJ280	Gohir.D06G031850.1.v3.1	ChrD06:3818632-3820120
GhDnaJ281	Gohir.D06G033400.1.v3.1	ChrD06:4025494-4032444
GhDnaJ282	Gohir.D06G033400.2.v3.1	ChrD06:4025494-4032409
GhDnaJ283	Gohir.D06G033400.3.v3.1	ChrD06:4025746-4032410
GhDnaJ284	Gohir.D06G033400.4.v3.1	ChrD06:4025746-4032410
GhDnaJ285	Gohir.D06G033400.5.v3.1	ChrD06:4025522-4032375
GhDnaJ286	Gohir.D06G037901.1.v3.1	ChrD06:4709753-4710343
GhDnaJ287	Gohir.D06G047700.1.v3.1	ChrD06:6991902-6995272
GhDnaJ288	Gohir.D06G058501.1.v3.1	ChrD06:9654439-9668730
GhDnaJ289	Gohir.D06G120600.1.v3.1	ChrD06:36677170-36685958

GhDnaJ290	Gohir.D06G177800.2.v3.1	ChrD06:61281065-61283683
GhDnaJ291	Gohir.D06G181100.1.v3.1	ChrD06:61906682-61909784
GhDnaJ292	Gohir.D06G181100.2.v3.1	ChrD06:61906682-61909784
GhDnaJ293	Gohir.D06G181100.3.v3.1	ChrD06:61906682-61909776
GhDnaJ294	Gohir.D06G181300.1.v3.1	ChrD06:61919289-61921235
GhDnaJ295	Gohir.D06G187500.1.v3.1	ChrD06:63048494-63056262
GhDnaJ296	Gohir.D06G187500.4.v3.1	ChrD06:63048492-63056257
GhDnaJ297	Gohir.D06G187500.5.v3.1	ChrD06:63048492-63056257
GhDnaJ298	Gohir.D06G187500.6.v3.1	ChrD06:63048913-63056257
GhDnaJ299	Gohir.D06G203700.1.v3.1	ChrD06:65215820-65221323
GhDnaJ300	Gohir.D07G055200.1.v3.1	ChrD07:6138122-6142665
GhDnaJ301	Gohir.D07G092400.1.v3.1	ChrD07:11680127-11683455
GhDnaJ302	Gohir.D07G117300.1.v3.1	ChrD07:16776667-16779319
GhDnaJ303	Gohir.D07G135600.1.v3.1	ChrD07:21215764-21224564
GhDnaJ304	Gohir.D07G171300.1.v3.1	ChrD07:35236623-35240208
GhDnaJ305	Gohir.D07G171300.2.v3.1	ChrD07:35236883-35240187
GhDnaJ306	Gohir.D07G201200.1.v3.1	ChrD07:52985459-52992346
GhDnaJ307	Gohir.D08G011800.1.v3.1	ChrD08:375664-378411
GhDnaJ308	Gohir.D08G011800.2.v3.1	ChrD08:375329-378166
GhDnaJ309	Gohir.D08G063000.1.v3.1	ChrD08:6884957-6891635
GhDnaJ310	Gohir.D08G139500.1.v3.1	ChrD08:47204267-47208316
GhDnaJ311	Gohir.D08G139500.2.v3.1	ChrD08:47204234-47208298
GhDnaJ312	Gohir.D08G223700.1.v3.1	ChrD08:64855135-64856850
GhDnaJ313	Gohir.D08G235750.1.v3.1	ChrD08:66096717-66098763
GhDnaJ314	Gohir.D08G240700.3.v3.1	ChrD08:66629736-66632371
GhDnaJ315	Gohir.D08G249900.1.v3.1	ChrD08:67624532-67628181
GhDnaJ316	Gohir.D08G249900.2.v3.1	ChrD08:67624417-67627892
GhDnaJ317	Gohir.D09G027800.1.v3.1	ChrD09:8868714-8871209
GhDnaJ318	Gohir.D09G027800.2.v3.1	ChrD09:8868760-8871062

GhDnaJ319	Gohir.D09G062300.1.v3.1	ChrD09:31820393-31823515
GhDnaJ320	Gohir.D09G100000.1.v3.1	ChrD09:38712700-38715690
GhDnaJ321	Gohir.D09G116700.1.v3.1	ChrD09:41217475-41230045
GhDnaJ322	Gohir.D09G124100.1.v3.1	ChrD09:42240309-42245472
GhDnaJ323	Gohir.D09G146300.1.v3.1	ChrD09:45081856-45085841
GhDnaJ324	Gohir.D09G162100.1.v3.1	ChrD09:46577538-46581580
GhDnaJ325	Gohir.D09G178900.1.v3.1	ChrD09:48422872-48428642
GhDnaJ326	Gohir.D10G015300.1.v3.1	ChrD10:1289822-1292787
GhDnaJ327	Gohir.D10G053500.1.v3.1	ChrD10:4939975-4942591
GhDnaJ328	Gohir.D10G053500.2.v3.1	ChrD10:4939975-4942591
GhDnaJ329	Gohir.D10G165700.1.v3.1	ChrD10:48659621-48664015
GhDnaJ330	Gohir.D10G181200.1.v3.1	ChrD10:54003365-54007853
GhDnaJ331	Gohir.D11G045200.1.v3.1	ChrD11:3730052-3731842
GhDnaJ332	Gohir.D11G056400.1.v3.1	ChrD11:4857214-4858200
GhDnaJ333	Gohir.D11G081300.2.v3.1	ChrD11:6790489-6793883
GhDnaJ334	Gohir.D11G115600.1.v3.1	ChrD11:10646504-10651734
GhDnaJ335	Gohir.D11G115700.1.v3.1	ChrD11:10664720-10669336
GhDnaJ336	Gohir.D11G151800.1.v3.1	ChrD11:15107169-15115684
GhDnaJ337	Gohir.D11G151800.2.v3.1	ChrD11:15107234-15115670
GhDnaJ338	Gohir.D11G151800.3.v3.1	ChrD11:15107234-15115670
GhDnaJ339	Gohir.D11G182300.1.v3.1	ChrD11:20306451-20307682
GhDnaJ340	Gohir.D11G189600.1.v3.1	ChrD11:21646471-21652469
GhDnaJ341	Gohir.D11G233100.1.v3.1	ChrD11:42028089-42032625
GhDnaJ342	Gohir.D11G247400.2.v3.1	ChrD11:54038420-54040907
GhDnaJ343	Gohir.D11G247400.3.v3.1	ChrD11:54038420-54040907
GhDnaJ344	Gohir.D11G247400.5.v3.1	ChrD11:54038420-54040907
GhDnaJ345	Gohir.D11G247400.6.v3.1	ChrD11:54038420-54040907
GhDnaJ346	Gohir.D11G247400.7.v3.1	ChrD11:54038420-54040908
GhDnaJ347	Gohir.D11G247400.8.v3.1	ChrD11:54039121-54040908

GhDnaJ348	Gohir.D11G275300.1.v3.1	ChrD11:61565472-61568205
GhDnaJ349	Gohir.D11G281600.1.v3.1	ChrD11:62641660-62643065
GhDnaJ350	Gohir.D12G015750.1.v3.1	ChrD12:1892175-1895737
GhDnaJ351	Gohir.D12G029700.1.v3.1	ChrD12:3862695-3866821
GhDnaJ352	Gohir.D12G086100.1.v3.1	ChrD12:15792411-15796428
GhDnaJ353	Gohir.D12G091700.1.v3.1	ChrD12:32549067-32557070
GhDnaJ354	Gohir.D12G129600.1.v3.1	ChrD12:44054330-44056669
GhDnaJ355	Gohir.D12G131900.1.v3.1	ChrD12:44326340-44328456
GhDnaJ356	Gohir.D12G185400.2.v3.1	ChrD12:53685225-53688730
GhDnaJ357	Gohir.D12G185400.3.v3.1	ChrD12:53685511-53688722
GhDnaJ358	Gohir.D12G185400.4.v3.1	ChrD12:53685452-53688780
GhDnaJ359	Gohir.D12G255700.3.v3.1	ChrD12:61377133-61382830
GhDnaJ360	Gohir.D13G009800.1.v3.1	ChrD13:858568-865158
GhDnaJ361	Gohir.D13G030833.1.v3.1	ChrD13:3159329-3163012
GhDnaJ362	Gohir.D13G049900.1.v3.1	ChrD13:6240434-6245087
GhDnaJ363	Gohir.D13G049900.3.v3.1	ChrD13:6240460-6245087
GhDnaJ364	Gohir.D13G125400.1.v3.1	ChrD13:43373196-43379828
GhDnaJ365	Gohir.D13G133066.1.v3.1	ChrD13:46983754-46984166
GhDnaJ366	Gohir.D13G158100.1.v3.1	ChrD13:53737516-53747783
GhDnaJ367	Gohir.D13G158100.5.v3.1	ChrD13:53738554-53747783
GhDnaJ368	Gohir.D13G158100.6.v3.1	ChrD13:53738553-53747783
GhDnaJ369	Gohir.D13G177900.1.v3.1	ChrD13:57587568-57590118
GhDnaJ370	Gohir.D13G205000.1.v3.1	ChrD13:61100896-61104037
GhDnaJ371	Gohir.D13G205000.2.v3.1	ChrD13:61100893-61104037
GhDnaJ372	Gohir.D13G220800.1.v3.1	ChrD13:63072525-63079991
