# Supplementary material for: Genome-Wide Identification of DnaJ Gene Family and VIGS Analysis Reveal the Function of GhDnaJ316 in Floral Development for Upland Cotton
Source: Plants (Basel). 2025 Nov 5;14(21):3380. doi: 10.3390/plants14213380 (PMC12609765; doi:10.3390/plants14213380)
Supplement: Supplementary file 1 [file plants-14-03380-s001.zip › Table S3.pdf]

Table S3 Physicochemical properties of DnaJ protein members in *Gossypium hirsutum*.

**Name**	**Molecular Weight**	**Theoretical pI**	**Instability Index**	**Aliphatic Index**	**Grand Average of Hydropathicity**
GhDnaJ01	38128	5.33	37.45	86.33	-0.495
GhDnaJ02	38128	5.33	37.45	86.33	-0.495
GhDnaJ03	38128	5.33	37.45	86.33	-0.495
GhDnaJ04	38128	5.33	37.45	86.33	-0.495
GhDnaJ05	38128	5.33	37.45	86.33	-0.495
GhDnaJ06	38171	6.06	36.17	85.18	-0.503
GhDnaJ07	38171	6.06	36.17	85.18	-0.503
GhDnaJ08	37661	5.99	29.67	87	-0.467
GhDnaJ09	37662	6.29	31.09	85.53	-0.48
GhDnaJ10	38268	6.43	31.79	83.92	-0.525
GhDnaJ11	38256	6.37	30.09	83.07	-0.504
GhDnaJ12	43651	5.25	39.54	77.16	-0.601
GhDnaJ13	43428	5.46	39.92	78.83	-0.593
GhDnaJ14	44666	5.34	45.15	77.15	-0.528
GhDnaJ15	44536	5.21	44.29	77.84	-0.518
GhDnaJ16	43683	6.06	46.14	77.34	-0.52
GhDnaJ17	43678	5.99	46.62	77.34	-0.518
GhDnaJ18	39667	6.69	45.44	83.46	-0.416
GhDnaJ19	39667	6.69	45.44	83.46	-0.416
GhDnaJ20	39667	6.69	45.44	83.46	-0.416
GhDnaJ21	133546	8.18	49.53	65.09	-0.686
GhDnaJ22	133378	7.34	49.87	65.41	-0.676
GhDnaJ23	141497	6.23	48.96	65.16	-0.629
GhDnaJ24	141203	6.32	48.51	65.54	-0.619
GhDnaJ25	154556	5.84	45.79	59.67	-0.712
GhDnaJ26	151735	6	44.49	59.71	-0.723
GhDnaJ27	155594	6.02	44.98	59.71	-0.718

GhDnaJ28	158288	6	45.28	59.94	-0.713
GhDnaJ29	154429	5.98	44.81	59.94	-0.718
GhDnaJ30	14394	10.33	49.73	78.08	-0.483
GhDnaJ31	14788	10.24	48.55	75.75	-0.493
GhDnaJ32	14692	10.24	45.41	75.75	-0.459
GhDnaJ33	24254	4.86	51.88	83.33	-0.394
GhDnaJ34	24297	4.94	53.61	81.55	-0.435
GhDnaJ35	32177	9.07	35.11	73.94	-0.807
GhDnaJ36	32321	9.07	36.59	71.94	-0.877
GhDnaJ37	63634	8.88	50.44	84.99	-0.348
GhDnaJ38	65086	9.1	44.47	88.8	-0.339
GhDnaJ39	64259	9.27	52.37	88.64	-0.335
GhDnaJ40	64277	9.17	51.53	88.3	-0.337
GhDnaJ41	29354	6.44	50.05	80.57	-0.287
GhDnaJ42	29472	6.89	56.03	80.19	-0.283
GhDnaJ43	28484	9.59	55.57	40.46	-1.358
GhDnaJ44	22150	9.78	57.87	34.28	-1.584
GhDnaJ45	28373	9.63	64.35	38.82	-1.365
GhDnaJ46	26391	9.38	61.99	39.77	-1.394
GhDnaJ47	28293	9.5	45.98	42.49	-1.308
GhDnaJ48	26451	9.62	47.49	39.82	-1.415
GhDnaJ49	28184	9.67	49.88	43.23	-1.362
GhDnaJ50	27922	9.67	50.05	45.09	-1.331
GhDnaJ51	28051	9.6	50.24	44.89	-1.34
GhDnaJ52	26318	9.55	47.87	41.58	-1.392
GhDnaJ53	37055	9.28	30.29	65.65	-0.703
GhDnaJ54	35011	8.77	33.99	68.08	-0.565
GhDnaJ55	34622	8.77	33.66	67.48	-0.58
GhDnaJ56	38119	7.97	52.53	65.07	-0.572
GhDnaJ57	38073	8.25	53.51	65.34	-0.564

GhDnaJ58	52465	9.39	58.75	81.04	-0.446
GhDnaJ59	52445	9.3	56.93	82.06	-0.413
GhDnaJ60	234932	5.55	47.05	97.93	-0.049
GhDnaJ61	278741	6.08	46.97	96.95	-0.04
GhDnaJ62	281731	5.75	45.71	97.03	-0.074
GhDnaJ63	177333	5.23	43.73	101.47	-0.015
GhDnaJ64	177333	5.23	43.73	101.47	-0.015
GhDnaJ65	173387	5.18	43.59	101.06	-0.022
GhDnaJ66	262498	5.63	45.73	97.68	-0.048
GhDnaJ67	258551	5.59	45.67	97.35	-0.053
GhDnaJ68	54338	4.82	61.96	54.77	-0.834
GhDnaJ69	163196	5.77	47.03	60.79	-1.048
GhDnaJ70	147118	4.71	46.8	55.49	-1.049
GhDnaJ71	146878	4.65	45.34	56.68	-1.036
GhDnaJ72	106931	6.05	49.24	50.21	-1.025
GhDnaJ73	106148	6.01	50.09	50.1	-1.034
GhDnaJ74	104942	5.89	54.02	49.77	-1.011
GhDnaJ75	104834	6.19	51.44	49.88	-1.009
GhDnaJ76	104834	6.19	51.44	49.88	-1.009
GhDnaJ77	104834	6.19	51.44	49.88	-1.009
GhDnaJ78	15964	6.48	36.83	54.1	-0.712
GhDnaJ79	28633	9.04	49.51	36.95	-1.203
GhDnaJ80	32681	9.34	41.07	44.09	-1.1
GhDnaJ81	32620	9.42	46.53	47.22	-1.07
GhDnaJ82	29647	8.87	65.92	44.82	-1.039
GhDnaJ83	29613	8.87	64.65	46.37	-1.035
GhDnaJ84	29613	8.87	64.65	46.37	-1.035
GhDnaJ85	29613	8.87	64.65	46.37	-1.035
GhDnaJ86	29613	8.87	64.65	46.37	-1.035
GhDnaJ87	70006	5.3	52.41	58.22	-0.973

GhDnaJ88	69543	5.36	54.75	58.29	-0.982
GhDnaJ89	67431	5.36	45.06	53.9	-1.208
GhDnaJ90	67442	5.3	47.38	52.91	-1.236
GhDnaJ91	67442	5.3	47.38	52.91	-1.236
GhDnaJ92	67442	5.3	47.38	52.91	-1.236
GhDnaJ93	74069	7.95	61.63	68.27	-0.957
GhDnaJ94	74069	7.95	61.63	68.27	-0.957
GhDnaJ95	74069	7.95	61.63	68.27	-0.957
GhDnaJ96	74069	7.95	61.63	68.27	-0.957
GhDnaJ97	74069	7.95	61.63	68.27	-0.957
GhDnaJ98	74333	8.36	60.87	67.34	-0.972
GhDnaJ99	74333	8.36	60.87	67.34	-0.972
GhDnaJ100	72146	8.5	62.67	69.04	-0.958
GhDnaJ101	73899	7.92	59.04	67.52	-0.968
GhDnaJ102	73899	7.92	59.04	67.52	-0.968
GhDnaJ103	73899	7.92	59.04	67.52	-0.968
GhDnaJ104	29523	9.04	51.91	65.24	-1.225
GhDnaJ105	29423	9.13	49.4	65.24	-1.21
GhDnaJ106	66967	9.29	54.48	59.06	-1.236
GhDnaJ107	67211	8.87	57.7	59.73	-1.235
GhDnaJ108	80643	8.98	36.7	72.59	-0.373
GhDnaJ109	80429	9.04	38.47	73	-0.385
GhDnaJ110	63313	8.85	47.4	75.61	-0.333
GhDnaJ111	63441	8.85	47.68	75.47	-0.339
GhDnaJ112	63555	8.83	46.85	74.95	-0.336
GhDnaJ113	63427	8.83	46.56	75.08	-0.331
GhDnaJ114	63466	8.98	52.8	76.85	-0.354
GhDnaJ115	48031	8.72	56.55	64.51	-0.665
GhDnaJ116	48160	8.72	56.89	64.35	-0.672
GhDnaJ117	53267	8.42	54.2	72.6	-0.433

GhDnaJ118	53395	8.42	54.52	72.44	-0.44
GhDnaJ119	63421	8.79	51.93	76.33	-0.356
GhDnaJ120	63293	8.79	51.66	76.47	-0.351
GhDnaJ121	31370	9.68	65.36	69.6	-0.719
GhDnaJ122	31412	9.7	62.08	70.66	-0.71
GhDnaJ123	31284	9.66	57.19	74.52	-0.793
GhDnaJ124	31046	9.69	56.14	73.2	-0.794
GhDnaJ125	59619	6.07	55.48	56.85	-0.757
GhDnaJ126	59636	6.07	52.05	55.58	-0.793
GhDnaJ127	73767	5.35	50.83	58.49	-0.768
GhDnaJ128	73517	5.13	51.33	58.59	-0.776
GhDnaJ129	73517	5.13	51.33	58.59	-0.776
GhDnaJ130	66502	6.06	56.44	61.9	-0.828
GhDnaJ131	66558	5.71	56.91	60.17	-0.851
GhDnaJ132	66558	5.71	56.91	60.17	-0.851
GhDnaJ133	77646	6.18	58.92	57.54	-0.862
GhDnaJ134	79350	6.6	57.03	54.12	-0.885
GhDnaJ135	79350	6.6	57.03	54.12	-0.885
GhDnaJ136	79350	6.6	57.03	54.12	-0.885
GhDnaJ137	37950	9.33	52.79	62.59	-0.694
GhDnaJ138	42293	9.69	53.81	62.39	-0.711
GhDnaJ139	38232	6.55	50.31	69.76	-0.645
GhDnaJ140	38216	6.95	50.11	69.76	-0.645
GhDnaJ141	38216	6.95	50.11	69.76	-0.645
GhDnaJ142	38216	6.95	50.11	69.76	-0.645
GhDnaJ143	38216	6.95	50.11	69.76	-0.645
GhDnaJ144	38149	8.94	51.93	70.96	-0.522
GhDnaJ145	38061	8.84	54.14	69.27	-0.556
GhDnaJ146	38061	8.84	54.14	69.27	-0.556
GhDnaJ147	37167	9.04	54.94	70.96	-0.611

GhDnaJ148	33365	8.97	53.16	72.75	-0.672
GhDnaJ149	36931	8.94	53.46	71.09	-0.627
GhDnaJ150	77707	8.86	44.75	65.8	-0.863
GhDnaJ151	73479	6.66	47.89	82.64	-0.29
GhDnaJ152	77965	7.52	49.34	79.77	-0.374
GhDnaJ153	78026	7.02	48.37	80.06	-0.375
GhDnaJ154	75853	8.83	32.72	65.81	-0.548
GhDnaJ155	75814	8.78	32.86	65.68	-0.55
GhDnaJ156	56436	9.14	32.86	68.64	-0.495
GhDnaJ157	77688	8.99	31.55	71.58	-0.474
GhDnaJ158	77795	8.99	29.69	71.71	-0.462
GhDnaJ159	56487	9.14	30.81	68.26	-0.482
GhDnaJ160	29673	5.51	39.15	75.43	-0.512
GhDnaJ161	84893	9.11	34.45	68.43	-0.626
GhDnaJ162	85840	9.24	36.84	65.71	-0.678
GhDnaJ163	89657	8.71	40.9	63.72	-0.752
GhDnaJ164	89657	8.71	40.9	63.72	-0.752
GhDnaJ165	89925	8.86	37.87	62.39	-0.774
GhDnaJ166	89925	8.86	37.87	62.39	-0.774
GhDnaJ167	78064	8.74	33.28	64.88	-0.817
GhDnaJ168	78183	8.67	34.56	64.13	-0.813
GhDnaJ169	79829	8.51	31.43	64.94	-0.808
GhDnaJ170	79889	8.79	31.98	65.47	-0.777
GhDnaJ171	79889	8.79	31.98	65.47	-0.777
GhDnaJ172	106919	8.35	43.42	61.53	-0.701
GhDnaJ173	106880	8.13	42.31	61.56	-0.704
GhDnaJ174	105671	6.33	43.06	59.4	-0.799
GhDnaJ175	105599	6.52	43.7	59.61	-0.804
GhDnaJ176	56334	8.56	50.89	49.7	-1.004
GhDnaJ177	130211	8.41	42.93	62.15	-0.761

GhDnaJ178	130261	8.45	43.9	62.69	-0.75
GhDnaJ179	130261	8.45	43.9	62.69	-0.75
GhDnaJ180	48584	9.45	39.72	50.66	-0.878
GhDnaJ181	39836	9.14	37.75	52.87	-0.845
GhDnaJ182	39836	9.14	37.75	52.87	-0.845
GhDnaJ183	39836	9.14	37.75	52.87	-0.845
GhDnaJ184	39803	9.22	36.59	53.4	-0.804
GhDnaJ185	39803	9.22	36.59	53.4	-0.804
GhDnaJ186	39803	9.22	36.59	53.4	-0.804
GhDnaJ187	39803	9.22	36.59	53.4	-0.804
GhDnaJ188	39803	9.22	36.59	53.4	-0.804
GhDnaJ189	39803	9.22	36.59	53.4	-0.804
GhDnaJ190	16807	5.75	47.55	62.64	-0.586
GhDnaJ191	16912	5.46	46	62.21	-0.6
GhDnaJ192	12120	10.28	30.92	64.73	-0.343
GhDnaJ193	12110	10.33	31.22	64.73	-0.339
GhDnaJ194	13552	10.9	55.63	62.93	-0.488
GhDnaJ195	13505	10.7	59.61	66.1	-0.464
GhDnaJ196	31485	5.78	65.63	58.96	-0.927
GhDnaJ197	32742	5.78	63.3	61.85	-0.88
GhDnaJ198	32655	5.63	63.05	61.85	-0.817
GhDnaJ199	31398	5.62	65.37	58.96	-0.862
GhDnaJ200	31398	5.62	65.37	58.96	-0.862
GhDnaJ201	19538	9.64	34.65	67.33	-0.742
GhDnaJ202	19559	9.76	37.34	66.73	-0.817
GhDnaJ203	19874	9.2	45.04	61.66	-0.829
GhDnaJ204	19829	9.32	45.13	62.25	-0.839
GhDnaJ205	19830	4.49	36.44	94.11	-0.233
GhDnaJ206	19830	4.49	36.44	94.11	-0.233
GhDnaJ207	19830	4.49	36.44	94.11	-0.233

GhDnaJ208	19789	4.49	30.01	93.54	-0.259
GhDnaJ209	19789	4.49	30.01	93.54	-0.259
GhDnaJ210	19789	4.49	30.01	93.54	-0.259
GhDnaJ211	19789	4.49	30.01	93.54	-0.259
GhDnaJ212	19789	4.49	30.01	93.54	-0.259
GhDnaJ213	19789	4.49	30.01	93.54	-0.259
GhDnaJ214	19789	4.49	30.01	93.54	-0.259
GhDnaJ215	19789	4.49	30.01	93.54	-0.259
GhDnaJ216	60006	7.92	41.63	76.52	-0.396
GhDnaJ217	60008	6.74	40.49	75.97	-0.407
GhDnaJ218	60008	6.74	40.49	75.97	-0.407
GhDnaJ219	22585	9.79	68.53	65.13	-1.002
GhDnaJ220	22423	9.59	58.18	63.07	-0.954
GhDnaJ221	32809	9.79	55.57	66.97	-1.057
GhDnaJ222	32783	9.79	55.57	65.91	-1.076
GhDnaJ223	29109	6.54	42.73	72.18	-0.65
GhDnaJ224	29106	6.98	43.82	72.18	-0.664
GhDnaJ225	16476	10.13	73.68	75.34	-0.382
GhDnaJ226	16310	9.92	66.91	76.01	-0.323
GhDnaJ227	15409	9.99	63.11	71.76	-0.34
GhDnaJ228	15404	9.86	66.47	66.76	-0.42
GhDnaJ229	18205	9.3	52.39	66.51	-0.302
GhDnaJ230	18296	9.12	61.32	63.61	-0.363
GhDnaJ231	12528	8.89	39.59	56.82	-0.663
GhDnaJ232	12529	8.89	41.76	58.64	-0.598
GhDnaJ233	17032	9.75	51.33	65.69	-0.579
GhDnaJ234	17032	9.75	51.33	65.69	-0.579
GhDnaJ235	17136	9.44	47.29	72.05	-0.287
GhDnaJ236	17183	9.78	50.73	71.41	-0.33
GhDnaJ237	17164	9.62	47.23	77.99	-0.249

GhDnaJ238	17460	9.68	48.61	75.44	-0.367
GhDnaJ239	47332	9.18	35.53	80	-0.351
GhDnaJ240	47429	9.31	36.66	79.31	-0.388
GhDnaJ241	46188	9.15	47.51	72.62	-0.317
GhDnaJ242	47909	9.39	43.9	74.79	-0.355
GhDnaJ243	47819	9.39	43.25	75.44	-0.342
GhDnaJ244	54129	8.88	36.92	68.28	-0.392
GhDnaJ245	54191	8.91	35.92	68.11	-0.408
GhDnaJ246	59685	9.31	40.82	72.56	-0.447
GhDnaJ247	45413	9.02	34.62	73.56	-0.36
GhDnaJ248	58767	9.29	40.9	74.69	-0.363
GhDnaJ249	37292	9.52	45.76	83.86	-0.27
GhDnaJ250	48376	9.04	36.09	73.49	-0.44
GhDnaJ251	48420	9.04	36.84	75.05	-0.421
GhDnaJ252	53503	7.14	40.33	72.35	-0.416
GhDnaJ253	53503	7.14	40.33	72.35	-0.416
GhDnaJ254	49555	6.8	41.26	65.48	-0.519
GhDnaJ255	50632	6.8	40.93	65.9	-0.516
GhDnaJ256	50632	6.8	40.93	65.9	-0.516
GhDnaJ257	53629	8.15	38.95	73.79	-0.392
GhDnaJ258	54787	8.13	38.12	75.47	-0.346
GhDnaJ259	51917	7.89	38.59	69.31	-0.44
GhDnaJ260	49681	7.91	39.78	67.02	-0.493
GhDnaJ261	50758	7.91	39.47	67.41	-0.491
GhDnaJ262	35358	9.24	48.23	91.07	-0.507
GhDnaJ263	35372	9.24	47.69	91.4	-0.512
GhDnaJ264	75572	5.6	47.16	95.35	-0.107
GhDnaJ265	66720	5.21	48.77	93.32	-0.217
GhDnaJ266	75712	5.6	46.53	94.67	-0.124
GhDnaJ267	75712	5.6	46.53	94.67	-0.124

GhDnaJ268	76353	5.47	50.24	89.11	-0.184
GhDnaJ269	76342	5.47	49.64	89.4	-0.19
GhDnaJ270	76342	5.47	49.64	89.4	-0.19
GhDnaJ271	76342	5.47	49.64	89.4	-0.19
GhDnaJ272	76342	5.47	49.64	89.4	-0.19
GhDnaJ273	36941	8.61	40.25	64.73	-0.775
GhDnaJ274	36855	8.62	38.25	64.73	-0.765
GhDnaJ275	37184	8.63	34.91	68.45	-0.722
GhDnaJ276	37265	8.97	36.28	69.41	-0.71
GhDnaJ277	37265	8.97	36.28	69.41	-0.71
GhDnaJ278	39930	9.15	42.89	73.59	-0.774
GhDnaJ279	39774	9.15	43.26	74.12	-0.753
GhDnaJ280	32050	9.37	42.71	84.6	-0.569
GhDnaJ281	32081	9.3	41.59	84.6	-0.571
GhDnaJ282	37319	9.23	35.33	65.97	-0.634
GhDnaJ283	37323	9.23	35.33	64.53	-0.648
GhDnaJ284	37808	9.1	39.98	62.83	-0.652
GhDnaJ285	37820	9.19	44.73	65.98	-0.633
GhDnaJ286	38561	8.99	34.8	78.99	-0.41
GhDnaJ287	38639	8.66	32.73	81.24	-0.395
GhDnaJ288	38004	9.13	38.1	67.76	-0.653
GhDnaJ289	37960	9.14	37.57	66.92	-0.667
GhDnaJ290	38899	6.4	40.7	74.94	-0.628
GhDnaJ291	38977	6.35	39.31	76.15	-0.595
GhDnaJ292	37810	6.58	41.11	72.03	-0.731
GhDnaJ293	38756	6.19	38.43	81.05	-0.507
GhDnaJ294	38771	6.11	36.39	79.33	-0.524
GhDnaJ295	38784	6.24	38.24	80.47	-0.516
GhDnaJ296	38860	6.05	37.99	80.17	-0.529
GhDnaJ297	47399	7.23	38.74	59.91	-0.85

GhDnaJ298	47213	6.97	37.27	61	-0.829
GhDnaJ299	46275	5.78	39.64	64.53	-0.731
GhDnaJ300	46231	5.68	38.59	64.53	-0.729
GhDnaJ301	46199	5.87	34.19	66.35	-0.684
GhDnaJ302	46214	5.96	33.64	66.8	-0.691
GhDnaJ303	46531	6.06	35.02	62.75	-0.756
GhDnaJ304	46506	6.12	31.43	64.14	-0.747
GhDnaJ305	46422	5.95	36.82	63.92	-0.727
GhDnaJ306	46551	6.06	35.63	62.06	-0.767
GhDnaJ307	46551	6.06	35.63	62.06	-0.767
GhDnaJ308	19464	10.15	44.78	62.06	-0.774
GhDnaJ309	20583	9.9	54.91	59.94	-0.766
GhDnaJ310	20618	9.79	55.02	57.24	-0.808
GhDnaJ311	64132	9.3	45.41	71.16	-0.476
GhDnaJ312	64237	9.3	46.58	70	-0.469
GhDnaJ313	61975	9.54	48.07	75.55	-0.458
GhDnaJ314	8655	9.6	25.44	92.5	-0.411
GhDnaJ315	65638	9.52	44.47	74.92	-0.454
GhDnaJ316	40830	8.89	52.26	71.16	-0.754
GhDnaJ317	40801	8.67	51.04	71.16	-0.752
GhDnaJ318	40801	8.67	51.04	71.16	-0.752
GhDnaJ319	42381	8.87	52.33	65.82	-0.576
GhDnaJ320	42381	8.87	52.33	65.82	-0.576
GhDnaJ321	24576	6.92	37.61	78.98	-0.386
GhDnaJ322	24450	8.36	36.53	78.98	-0.352
GhDnaJ323	41697	8.87	54.72	65.89	-0.595
GhDnaJ324	41697	8.87	54.72	65.89	-0.595
GhDnaJ325	53283	7.17	42.16	86.46	-0.381
GhDnaJ326	53711	6.85	39.55	89.54	-0.35
GhDnaJ327	54534	6.88	40.22	87.9	-0.365

GhDnaJ328	54581	6.53	38.97	88.13	-0.365
GhDnaJ329	59878	8.86	31.48	87.1	-0.375
GhDnaJ330	59928	8.86	32.74	88.89	-0.365
GhDnaJ331	45304	5.86	36.28	71.11	-0.566
GhDnaJ332	45374	6	37.46	72.07	-0.543
GhDnaJ333	46185	8.48	39.69	73.03	-0.6
GhDnaJ334	45440	8.03	39.14	73.81	-0.606
GhDnaJ335	46342	8.3	37.98	74.02	-0.584
GhDnaJ336	46781	7.61	46.12	70.95	-0.628
GhDnaJ337	44961	7.58	45.17	69.87	-0.665
GhDnaJ338	46752	7.09	43.03	70	-0.637
GhDnaJ339	33070	8.16	48.88	58.15	-1.146
GhDnaJ340	33070	8.16	48.88	58.15	-1.146
GhDnaJ341	33162	8.16	49.64	57.14	-1.175
GhDnaJ342	28604	5.53	39.73	69.56	-0.902
GhDnaJ343	32179	6.22	46.33	64.11	-0.994
GhDnaJ344	12694	5.31	19.5	77.89	-0.721
GhDnaJ345	14501	4.84	22.05	72.42	-0.822
GhDnaJ346	14501	4.84	22.05	72.42	-0.822
GhDnaJ347	14885	5.37	19.03	73.57	-0.909
GhDnaJ348	25328	6.96	31.61	87.06	-0.452
GhDnaJ349	15776	4.71	56.54	82.11	-0.778
GhDnaJ350	15807	4.66	58.41	81.35	-0.783
GhDnaJ351	12091	4.66	66.79	60.58	-1.05
GhDnaJ352	23090	4.76	78.32	53.16	-0.881
GhDnaJ353	23135	4.93	76.32	46.5	-0.969
GhDnaJ354	25674	4.86	73.23	52.24	-0.852
GhDnaJ355	25712	5.13	71.09	46.77	-0.918
GhDnaJ356	25574	5.37	63.19	41.35	-0.897
GhDnaJ357	23592	5.23	66.06	39.44	-0.901

GhDnaJ358	23607	5.13	66.46	38.97	-0.914
GhDnaJ359	26111	5.67	59.64	42.85	-0.824
GhDnaJ360	26054	6.13	59.15	42.47	-0.819
GhDnaJ361	28036	6.24	57.01	44	-0.82
GhDnaJ362	19982	4.82	67.7	55.26	-0.796
GhDnaJ363	19795	5.52	49.92	80.65	-0.335
GhDnaJ364	11878	5.79	38.98	66.31	-0.633
GhDnaJ365	16912	5.3	50.44	61.51	-0.758
GhDnaJ366	16821	5.53	51.48	62.19	-0.782
GhDnaJ367	18081	5.11	49.26	48.86	-0.823
GhDnaJ368	18067	4.98	48.98	45.76	-0.822
GhDnaJ369	16954	4.89	31	59.47	-0.586
GhDnaJ370	16994	4.87	35.15	50.46	-0.67
GhDnaJ371	16994	4.87	35.15	50.46	-0.67
GhDnaJ372	16994	4.87	35.15	50.46	-0.67
