# Supplementary material for: Genome-Wide Identification of DnaJ Gene Family and VIGS Analysis Reveal the Function of GhDnaJ316 in Floral Development for Upland Cotton
Source: Plants (Basel). 2025 Nov 5;14(21):3380. doi: 10.3390/plants14213380 (PMC12609765; doi:10.3390/plants14213380)
Supplement: Supplementary file 1 [file plants-14-03380-s001.zip › Table S4.pdf]

Table S4 Statistics of relative expression in empty vector and silenced plants.

**empty vector or silenced samples**	**relative expression**
TRV:00	1.000000909
TRV:00	0.857906223
TRV:00	1.165627506
TRV:GhDnaJ316 (1)	0.385213607
TRV:GhDnaJ316 (1)	0.420589117
TRV:GhDnaJ316 (1)	0.390360871
TRV:GhDnaJ316 (2)	0.485295441
TRV:GhDnaJ316 (2)	0.598527855
TRV:GhDnaJ316 (2)	0.629005119
TRV:GhDnaJ316 (3)	0.672030015
TRV:GhDnaJ316 (3)	0.403771312
TRV:GhDnaJ316 (3)	0.488814036
TRV:GhDnaJ316 (4)	0.430148725
TRV:GhDnaJ316 (4)	0.390914488
TRV:GhDnaJ316 (4)	0.355257968
