# Supplementary material for: Genome-Wide Identification of DnaJ Gene Family and VIGS Analysis Reveal the Function of GhDnaJ316 in Floral Development for Upland Cotton
Source: Plants (Basel). 2025 Nov 5;14(21):3380. doi: 10.3390/plants14213380 (PMC12609765; doi:10.3390/plants14213380)
Supplement: Supplementary file 1 [file plants-14-03380-s001.zip › Table S5.pdf]

Table S5 Statistics of budding time in empty vector and silenced plants.

**empty vector or silenced samples**	**budding time (d)**
TRV:00	65
TRV:00	65
TRV:00	68
TRV:00	64
TRV:00	73
TRV:00	68
TRV:00	77
TRV:GhDnaJ316	53
TRV:GhDnaJ316	57
TRV:GhDnaJ316	63
TRV:GhDnaJ316	66
TRV:GhDnaJ316	60
TRV:GhDnaJ316	64
TRV:GhDnaJ316	63
