# Supplementary material for: Genome-Wide Identification of DnaJ Gene Family and VIGS Analysis Reveal the Function of GhDnaJ316 in Floral Development for Upland Cotton
Source: Plants (Basel). 2025 Nov 5;14(21):3380. doi: 10.3390/plants14213380 (PMC12609765; doi:10.3390/plants14213380)
Supplement: Supplementary file 1 [file plants-14-03380-s001.zip › Table S6.pdf]

Table S6 Statistics of flowering time in empty vector and silenced plants.

**empty vector or silenced samples**	**flowering time (d)**
TRV:00	79
TRV:00	76
TRV:00	62
TRV:00	77
TRV:00	81
TRV:00	80
TRV:00	86
TRV:00	76
TRV:00	70
TRV:00	79
TRV:GhDnaJ316	62
TRV:GhDnaJ316	56
TRV:GhDnaJ316	70
TRV:GhDnaJ316	64
TRV:GhDnaJ316	60
TRV:GhDnaJ316	62
TRV:GhDnaJ316	76
TRV:GhDnaJ316	65
TRV:GhDnaJ316	76
TRV:GhDnaJ316	74
