# Supplementary figures and images for: Genome-Wide Identification of DnaJ Gene Family and VIGS Analysis Reveal the Function of GhDnaJ316 in Floral Development for Upland Cotton
Source: Plants (Basel). 2025 Nov 5;14(21):3380. doi: 10.3390/plants14213380 (PMC12609765; doi:10.3390/plants14213380)

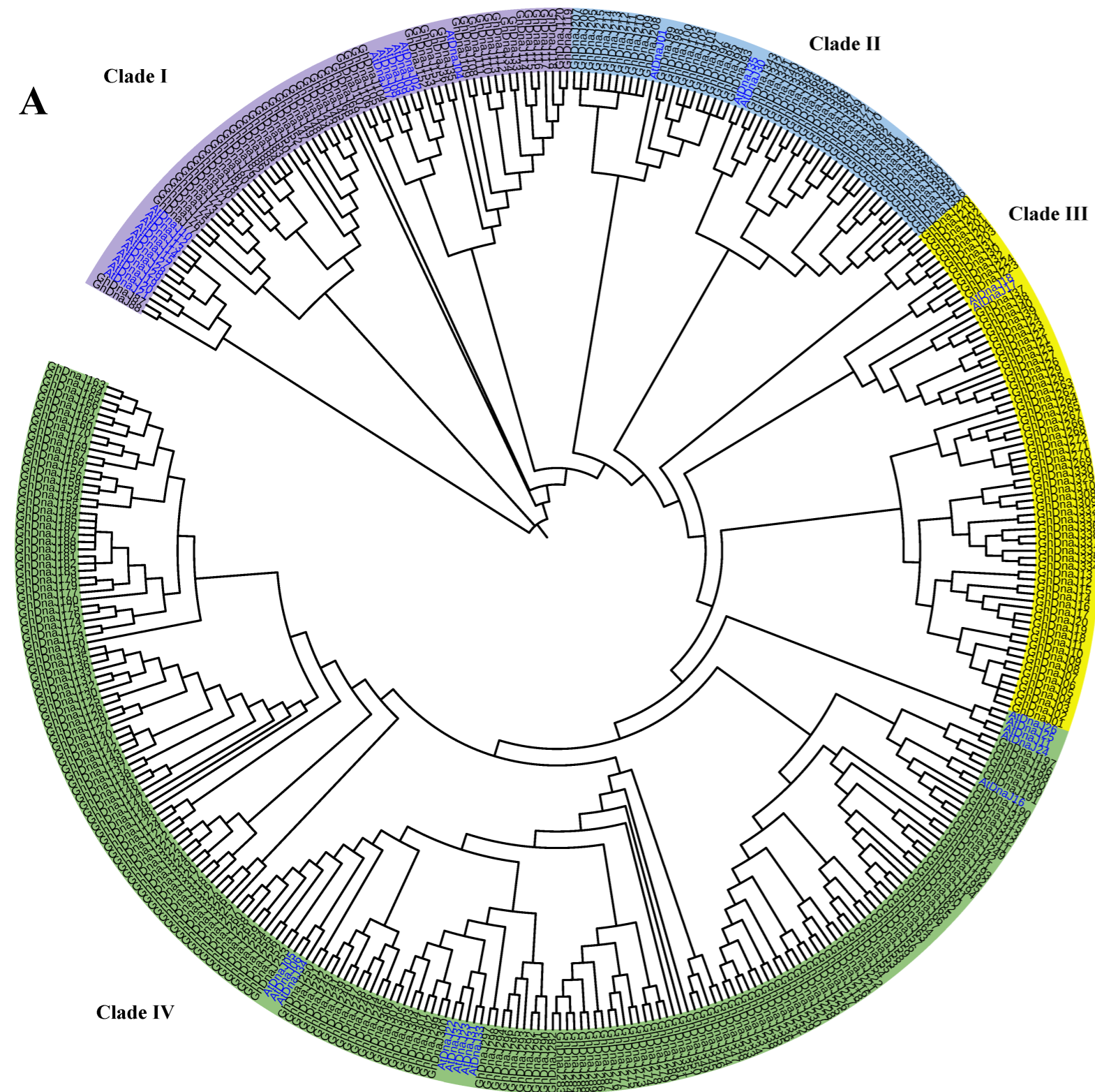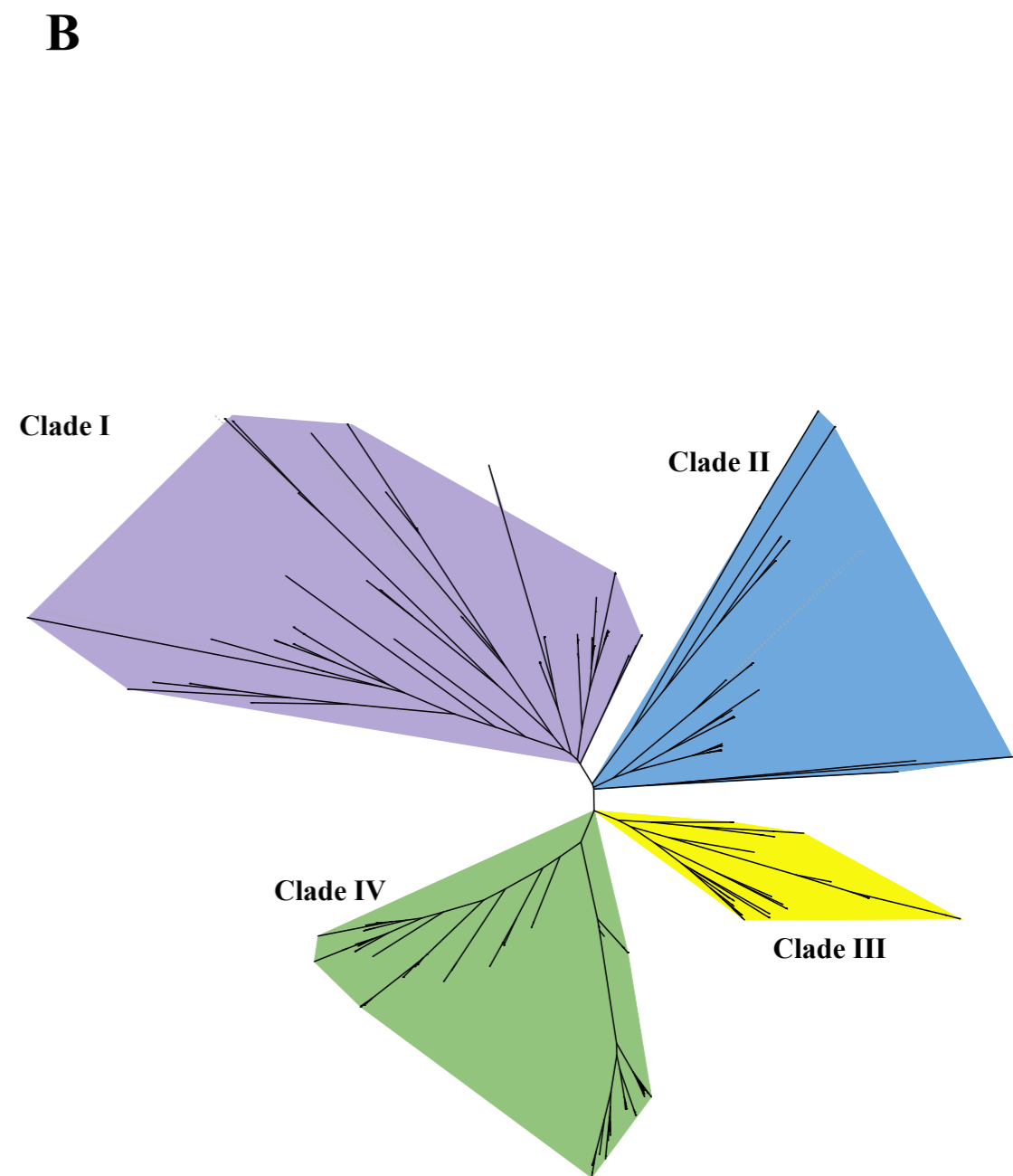

Supplement: Supplementary file 1 [file plants-14-03380-s001.zip › Fig. S2.pdf]

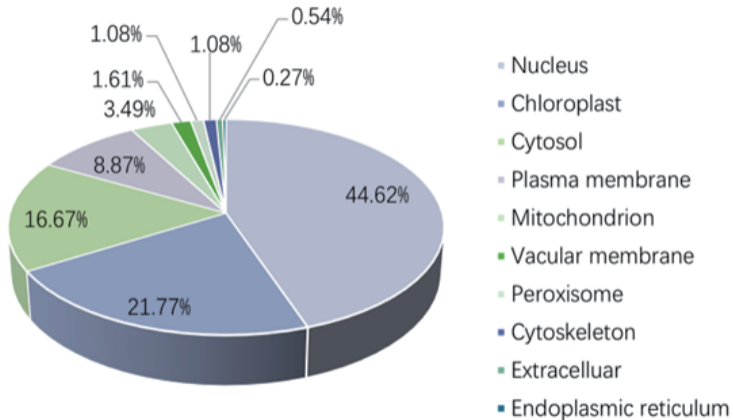

Supplement: Supplementary file 1 [file plants-14-03380-s001.zip › Fig.S1.pdf]

Clade I

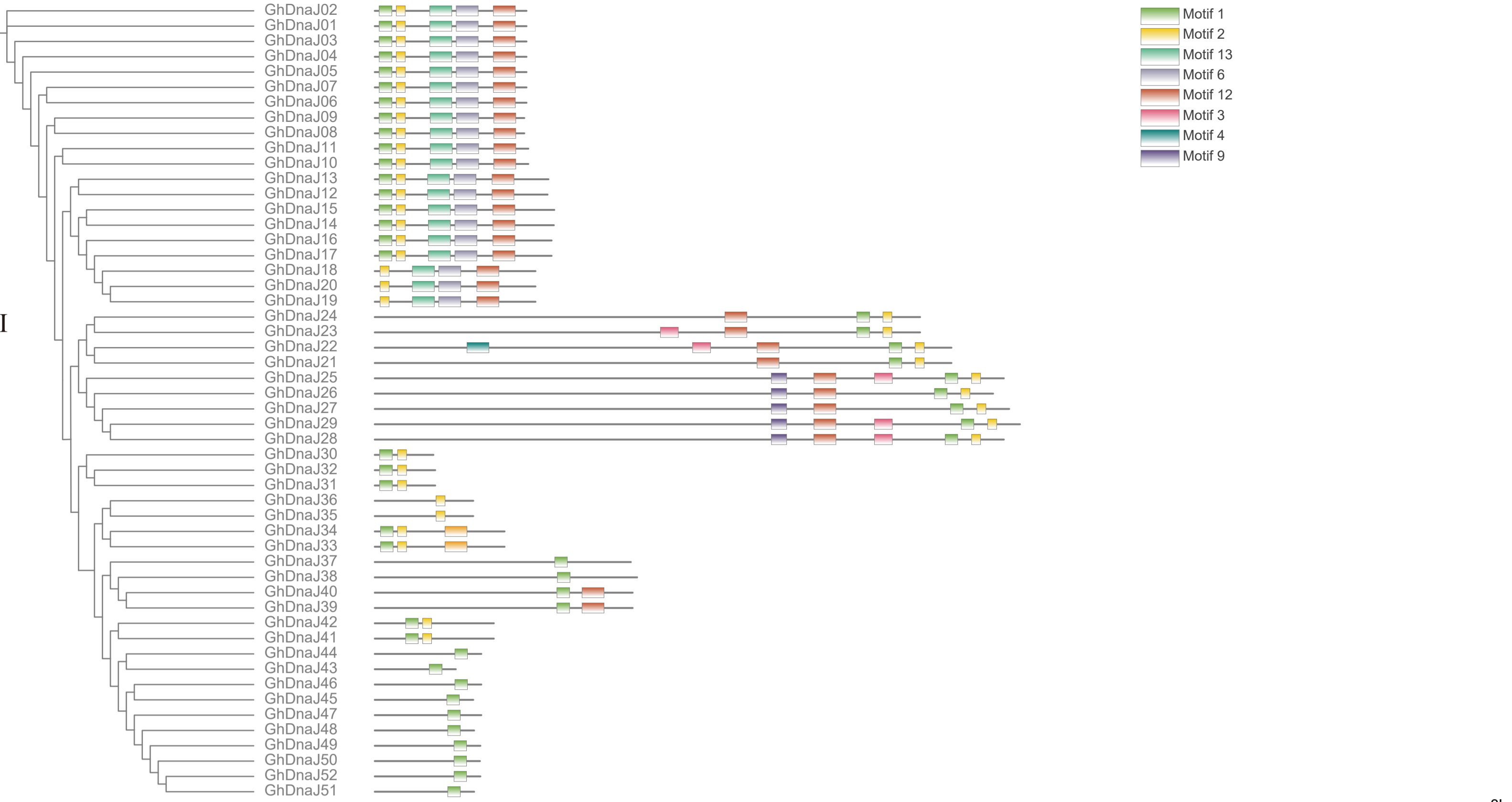

Clade II

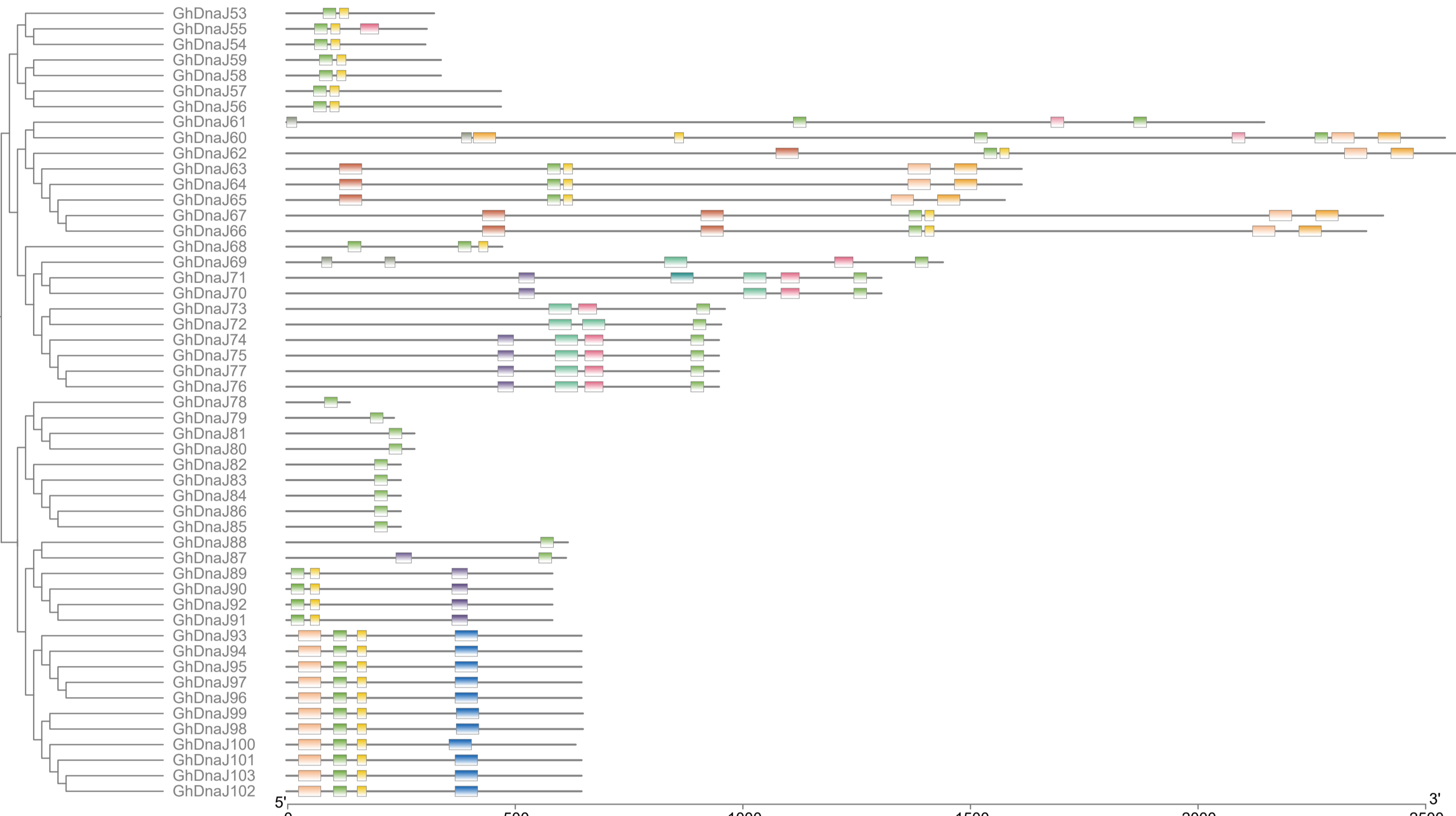

Clade III

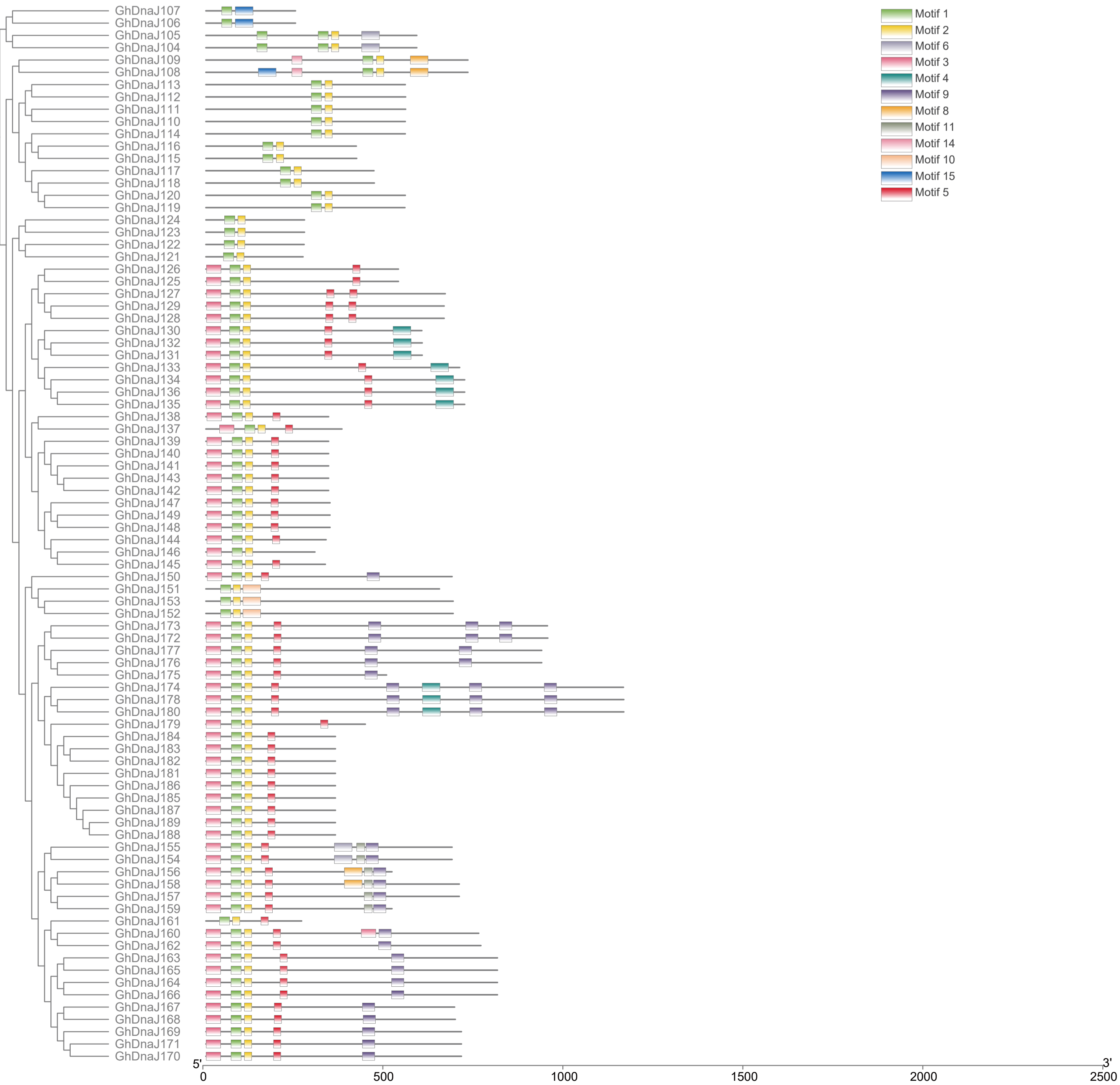

Clade IV

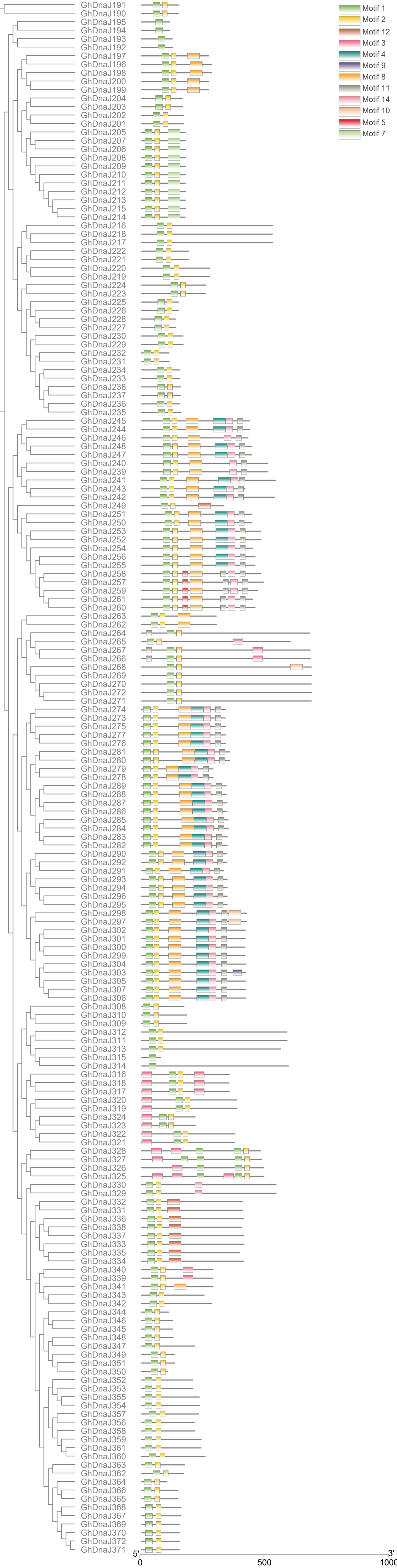

Supplement: Supplementary file 1 [file plants-14-03380-s001.zip › Fig.S3.pdf]

Clade I

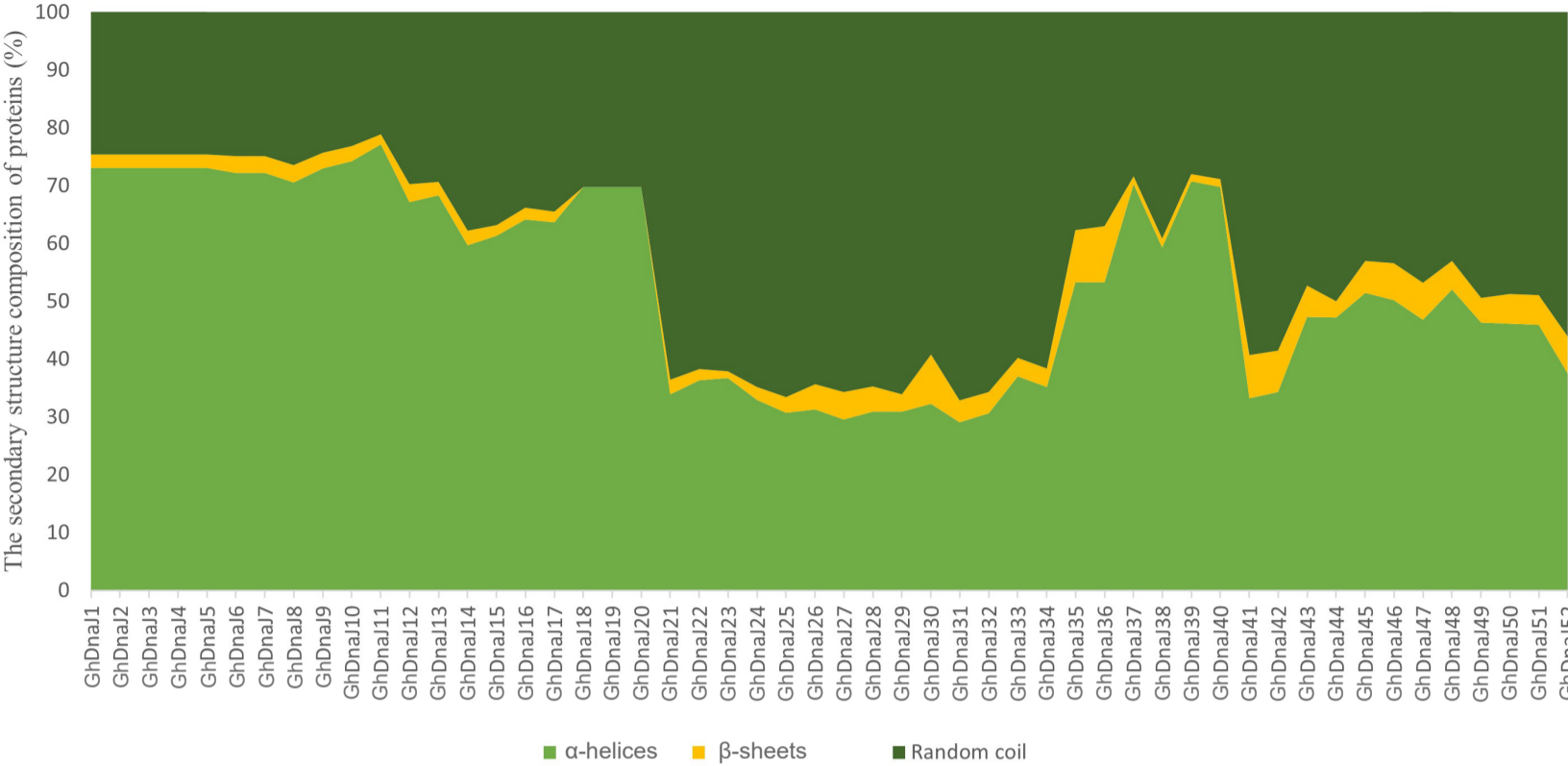

Clade II

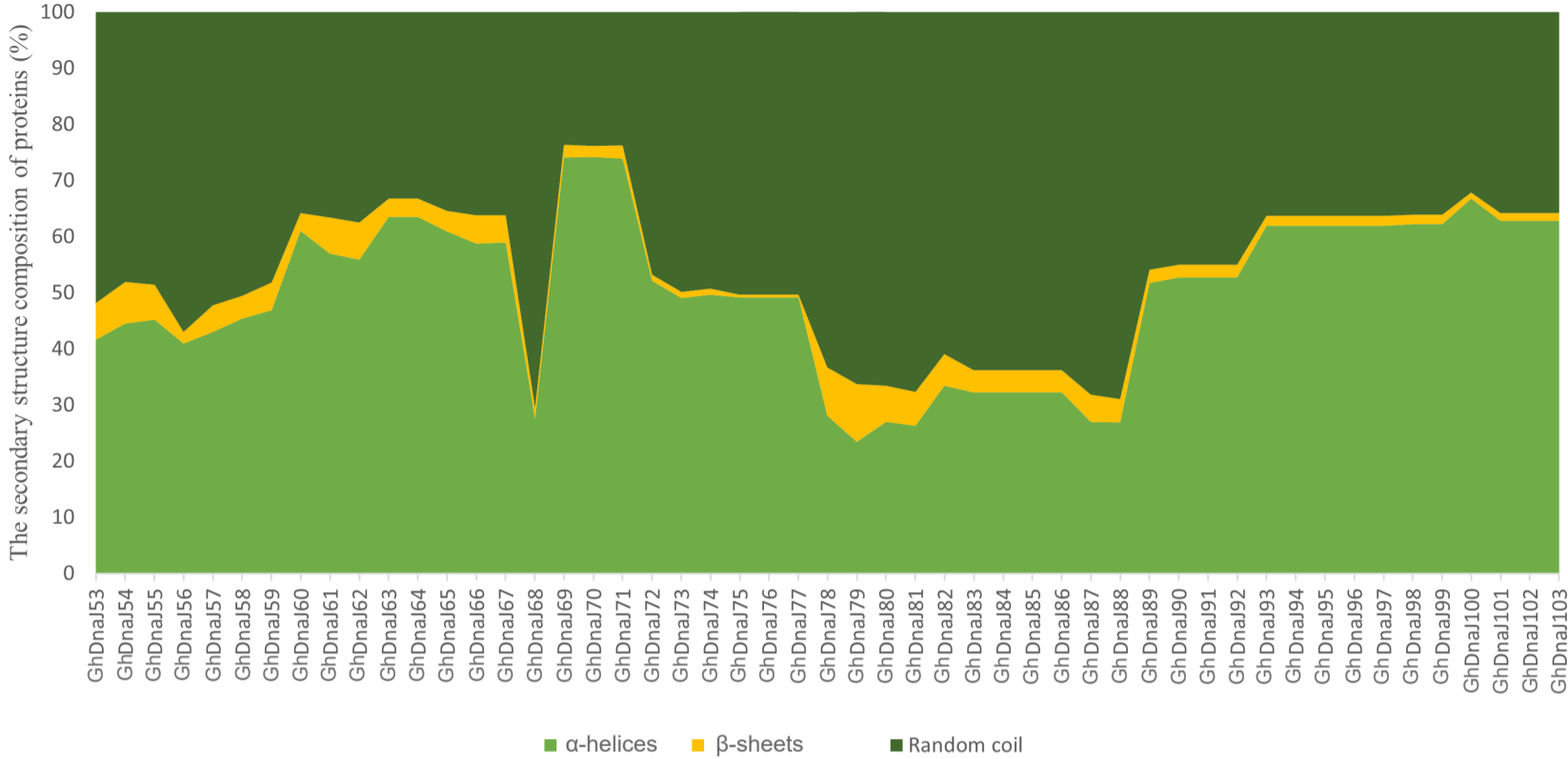

Clade III

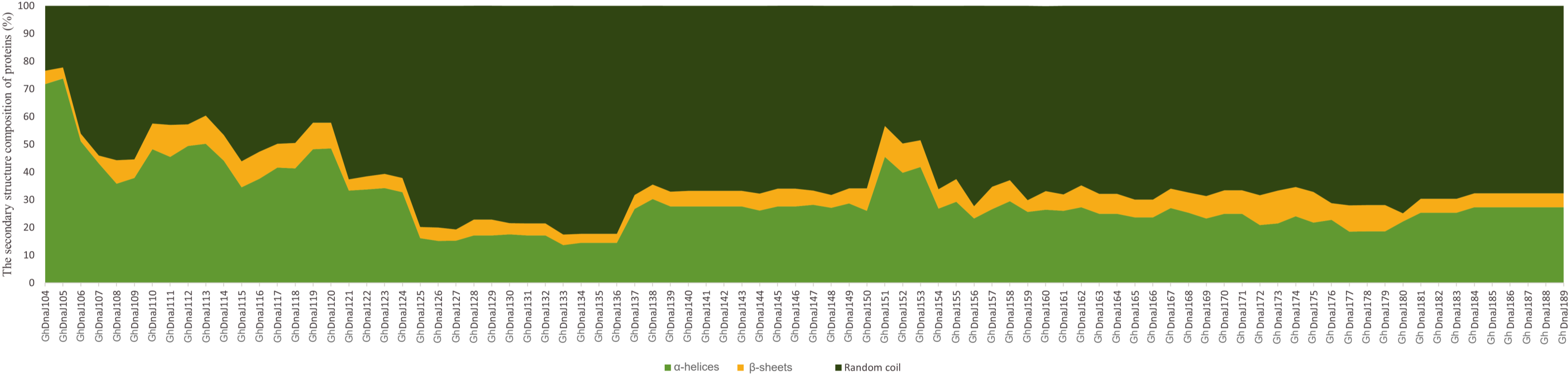

Clade IV

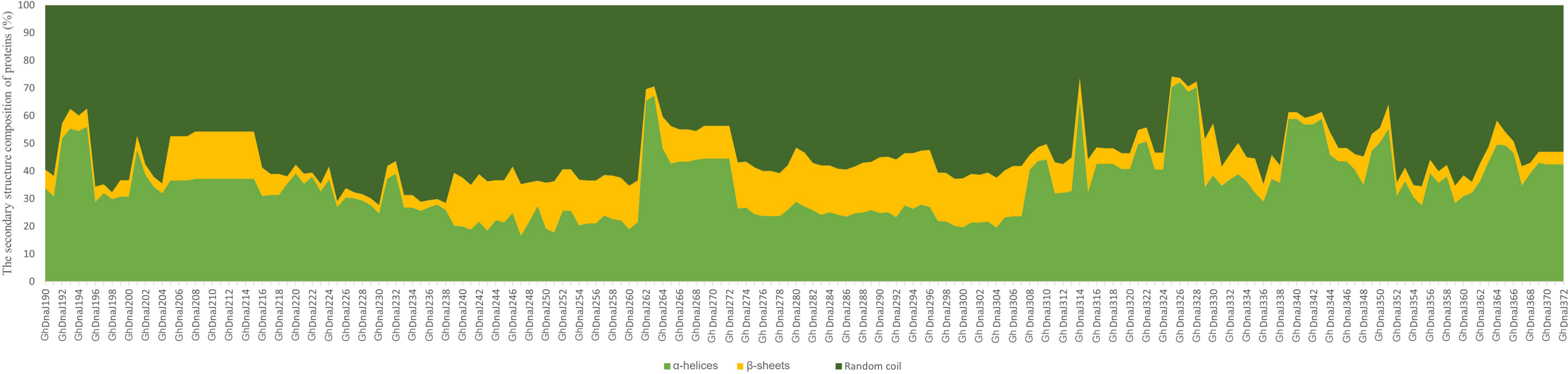

Supplement: Supplementary file 1 [file plants-14-03380-s001.zip › Fig.S4.pdf]

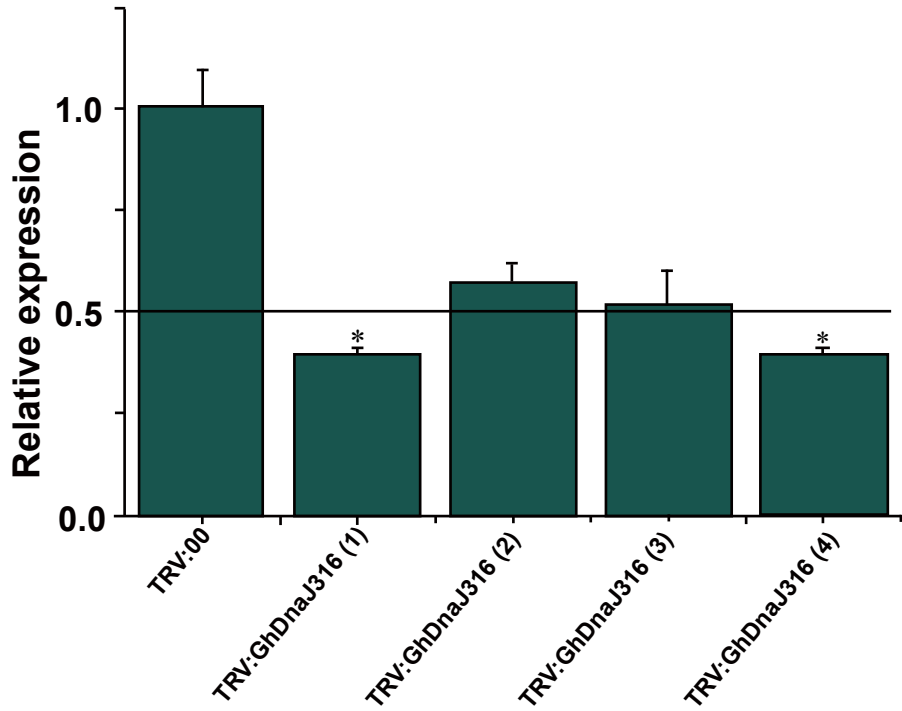

Supplement: Supplementary file 1 [file plants-14-03380-s001.zip › Fig.S7.pdf]
